# Mutational and Topological Analysis of the *Escherichia coli* BamA Protein

**DOI:** 10.1371/journal.pone.0084512

**Published:** 2013-12-23

**Authors:** Douglas F. Browning, Sophie A. Matthews, Amanda E. Rossiter, Yanina R. Sevastsyanovich, Mark Jeeves, Jessica L. Mason, Timothy J. Wells, Catherine A. Wardius, Timothy J. Knowles, Adam F. Cunningham, Vassiliy N. Bavro, Michael Overduin, Ian R. Henderson

**Affiliations:** 1 Institute of Microbiology and Infection, School of Biosciences, University of Birmingham, Birmingham, United Kingdom; 2 School of Cancer Sciences, University of Birmingham, Edgbaston, Birmingham, United Kingdom; University of Groningen, Groningen Institute for Biomolecular Sciences and Biotechnology, Netherlands

## Abstract

The multi-protein β-barrel assembly machine (BAM) of *Escherichia coli* is responsible for the folding and insertion of β-barrel containing integral outer membrane proteins (OMPs) into the bacterial outer membrane. An essential component of this complex is the BamA protein, which binds unfolded β-barrel precursors via the five polypeptide transport-associated (POTRA) domains in its N-terminus. The C-terminus of BamA contains a β-barrel domain, which tethers BamA to the outer membrane and is also thought to be involved in OMP insertion. Here we mutagenize BamA using linker scanning mutagenesis and demonstrate that all five POTRA domains are essential for BamA protein function in our experimental system. Furthermore, we generate a homology based model of the BamA β-barrel and test our model using insertion mutagenesis, deletion analysis and immunofluorescence to identify β-strands, periplasmic turns and extracellular loops. We show that the surface-exposed loops of the BamA β-barrel are essential.

## Introduction

The outer membranes of Gram-negative bacteria serve as a barrier to protect cells from toxic compounds such as antibiotics and detergents. They are composed of phospholipids, lipopolysaccharide and two major classes of proteins, lipoproteins and β-barrel containing integral outer membrane proteins (OMPs). In *Escherichia coli*, OMPs are expressed in the cytosol, transported across the inner membrane and periplasm, to be inserted and folded into the outer membrane. OMP insertion is achieved by the multi-protein β-barrel assembly machine (BAM) complex, which consists of the essential OMP BamA and four accessory lipoproteins (BamB, BamC, BamD and BamE) [1], [2]. This is an evolutionary conserved molecular machine, components of which are found in all Gram-negative bacteria as well as eukaryotic mitochondria and chloroplasts [3], [4].

BamA is an essential protein in *E. coli* and belongs to the Omp85 family of proteins [3], [5]. It consists of an N-terminal periplasmic domain composed of five polypeptide transport–associated (POTRA) motifs (POTRA_1_ to POTRA_5_) and a C-terminal β–barrel domain, which anchors the protein in the outer membrane [1], [2]. Deletion analysis of BamA POTRA domains suggested that POTRA_3_, POTRA_4_ and POTRA_5_ are essential for function but that POTRA_1_ and POTRA_2_ are dispensable, although cells expressing these deletion proteins grew extremely poorly [6], [7]. Various structures of the BamA POTRA domains have been determined and show that all five POTRA domains possess the same basic fold, comprising of a three stranded β-sheet associated with two α-helices [6], [8]–[11]. Elegant work by Kim *et al*. [6] demonstrated that BamD binds directly to BamA through POTRA_5_, whilst BamB requires POTRA_2_ to POTRA_5_. BamC and BamE do not bind to BamA directly but associate with the BAM complex through BamD [6]. In addition to their role of scaffolding the BAM lipoproteins, POTRA domains also bind unfolded OMPs and are thought to be responsible for delivering them to the outer membrane for insertion [6], [9], [10], [12].

The role that the C-terminal β-barrel of BamA plays in OMP insertion is less clear. Based on homology with the distant Omp85 family member FhaC from *Bordetella pertussis*, it has been proposed that the BamA C-terminal domain folds into a similar 16-stranded β-barrel [13]. Interestingly, the crystal structure of the FhaC β-barrel reveals a lumen that is in part occluded by a long external loop L6, which is essential for FhaC function [13]. A conserved sequence motif at the tip of this loop is also found in other Omp85 members, including BamA [14] and mutation of this “RGF” motif disrupts BamA function. This has led to speculation that a similar loop arrangement occurs in BamA [15], [16].

To gain insight into the organisation of BamA we have carried out systematic linker scanning mutation of BamA to identify regions crucial for function. In addition, we present a homology-based model of the BamA β-barrel and use insertion and deletion analysis to validate our topological predictions. Using this approach, we demonstrate that all five POTRA domains are essential for normal laboratory growth and viability and that the BamA β-barrel is integrally involved in OMP biogenesis, with external surface loops being critically important. Note, whilst this manuscript was under review Noinaj *et al.* [17] reported the crystal structures of the BamA homologues from *Neisseria gonorrhoeae* and *Haemophilus ducreyi*, revealing that the C-terminal domain of each protein adopts a 16-stranded β-barrel, over which the extracellular loops form a dome.

## Materials and Methods

### Bacterial strains, growth conditions, plasmids and primers

The bacterial strains and plasmids used in this study are listed in Table S1 and the primers used are detailed in Table S2. Strains were cultured in lysogeny broth (LB) [18], and Lennox broth (2% (w/v) peptone (Merck), 1% (w/v) yeast extract (Fisher Scientific) and 170 mM NaCl) [19], where stated, and on nutrient agar (Oxoid), LB agar [18] and M9 minimal agar containing 0.2% glucose [18]. Ampicillin (100 μg ml^−1^), kanamycin (50 µg ml^−1^) and vancomycin (37.5, 75 and 150 µg ml^−1^) were included in media where appropriate. To determine the ability of plasmid constructs to rescue BamA depletion on solid media, the *E. coli* BamA depletion strain JWD3 was grown on agar plates in the presence or absence of 0.2% (w/v) arabinose [20]. To assess this in liquid media, JWD3 cells were grown in 50 ml of Lennox broth at 37°C with shaking in the presence of 0.05% (w/v) arabinose or fructose and optical density (OD_600_) was monitored over time. After 300 minutes growth, cultures were sampled and subcultured into fresh medium. If constructs failed to rescue depletion in the presence of fructose, no further growth was detected after this point.

### Plasmid construction

The DNA encoding the full length *E. coli* K-12 BamA protein was synthesized by Genscript (www.genscript.com) and cloned into pET17b using *Nde*I and *Xho*I, to generate pET17b/*bamA*. To aid *bamA* manipulation, the gene was codon optimised for high level expression in *E. coli*, purged of restriction sites and unique sites for *Nde*I, *Nhe*I, *Bam*HI and *Xho*I were introduced (Fig. S1). Note that the *Nhe*I site introduces an additional serine after the *bamA* signal sequence (Fig. S2), however, this does not alter the ability of this BamA construct to function in *E. coli* (Fig. S3). To avoid confusion with other studies, all positions within BamA are denoted with respect to the *E. coli* BamA wild-type sequence, which lacks this additional serine. To introduce an N-terminal hexahistidine (6His) epitope tag on to BamA, plasmid pET17b/*6hisbamA* was generated by PCR using primers 6HisBamA and BamA1372Rev, with pET17b/*bamA* as template. PCR product was restricted with *Nhe*I and *Bam*HI and cloned into pET17b/*bamA*. This places the 6His tag directly after the BamA signal sequence.

Deletion of POTRA_1_ and POTRA_5_ was carried out using PCR with primer pairs PDΔ1 and BamA1372Rev, and PetPro and PDΔ5, with pET17b/*bamA* as template. Deletion of POTRA_2_, POTRA_3_ and POTRA_4_ was achieved by “megaprimer” PCR [21], [22]. *bamA* DNA was amplified using primer BamA1372Rev and primers PDΔ2, PDΔ3 and PDΔ4 with pET17b/*bamA* as template. PCR products were used in a second round of PCR with primer PetPro and pET17b/*bamA* as template. Constructs were cloned into pET17b/*bamA* using *Nde*I and *BamH*I and verified by DNA sequencing. The amino acid sequence of each POTRA deletion was identical to the POTRA deletion constructs generated by Kim *et al.* [6] (see Table S1).

Deletion of BamA loops L3, L4, L6, L7 and L8 was achieved using megaprimer PCR [21], [22]. DNA was amplified using pET17b/*bamA* as template and primer PetTerm with primer ΔL3, ΔL4, ΔL6, ΔL7 or ΔL8. PCR products were used in a second round of PCR with primer BamA1130Fw and pET17b/*bamA* as template. Final products were cloned into pET17b/*6hisbamA* using *BamH*I and *Xho*I and verified by DNA sequencing (see Table S1).

The insertion of an HA (human influenza hemagglutinin) epitope tag into β-strand β1 was generated by conventional PCR using primers β1HAFw and XhoIRev, with pET17b/*bamA* as template. The insertion of HA epitopes into other regions of BamA was achieved using megaprimer PCR [21], [22]. Primers L1HARev to L5HARev and β2HARev to β11HARev were used with primer BamHIFw to generate the megaprimer, which was then used with XhoIRev to generate the full length product. Primers L6HAFw to L8HAFw and β12HAFw to β16HAFw were used with XhoIRev to generate the initial megaprimer PCR and then primer BamHIFw was used to produce the completed PCR product. All DNA fragments were cloned into pET17b/*bamA* using *Bam*HI and *Xho*I and verified by DNA sequencing (see Table S1).

### Generation of the BamA linker scanning library

The BamA linker scanning library was generated using the Thermo Scientific Mutation Generation System Kit. Entranceposon M1-Kan^R^ was randomly introduced into plasmid pET17b/*bamA*, as specified by the manufacturers, and transformed into *E. coli* K-12 strain RLG221. Insertions were selected for by plating cells onto LB agar containing 50 µg ml^−1^ kanamycin and then screened for insertions within *bamA*. The entranceposon was removed by digesting each plasmid with *Not*I. Restricted plasmids were re-circularised using T4 DNA ligase and transformed into cells selecting for ampicillin resistance. The location of each 15 bp insertion within *bamA* was identified by DNA sequencing and all insertions are listed in Table S3. The position of each insertion is given as the last codon in BamA that was unaltered by the entranceposon insertion. Note that for all entranceposon insertions that were generated we were able to isolate the corresponding 15 bp insertion construct. 6His tagged versions of POTRA insertions were generated by PCR using primers 6HisBamA and BamA1372Rev and the relevant pET17b/*bamA* insertion construct as template. PCR product was restricted with *Nhe*I and *Bam*HI and cloned into pET17b/*bamA*. 6His tagged versions of barrel insertions were constructed by sub-cloning the *Nhe*I and *Bam*HI fragment from pET17b/*6hisbamA* into each pET17b/*bamA* insertion construct (see Table S4).

### Sample preparation and Western blotting

JWD3 cells, carrying the wild-type and mutant versions of pET17b/*bamA* and pET17b/*6hisbamA*, were grown in Lennox broth at 37°C with shaking for 5 h in the presence of 0.05% (w/v) arabinose or fructose. The preparation of normalised total cellular protein samples, isolation of membrane fractions and washing of membranes with urea were carried out as detailed [23]–[26]. Briefly, cells were isolated by centrifugation and pellets were washed with 10 mM Tris-HCl (pH 7.4) and resuspended in 20 ml of 10 mM Tris-HCl (pH 7.4) containing 2 mM PMSF. Cell envelopes were disrupted by sonication, using a Misonix XL sonicator, and unbroken cells were removed by centrifugation for 10 min at 6,000× g and 4°C. The total membrane fraction was then isolated by centrifuging the supernatant for 1 h at 48,000× g at 4°C, after which membranes were washed and resuspended in 1 ml of 10 mM Tris-HCl (pH 7.4). To isolate the outer membrane fraction, total membrane pellets were resuspended in 10 ml of 10 mM Tris-HCl (pH 7.4) containing 2% (v/v) Triton X-100 and incubated at 25°C for 15 min. The outer membrane containing fraction was isolated by centrifuging preparations for 1 h at 48,000× g and 4°C. The pelleted material was then washed three times and resuspended in 1 ml of 10 mM Tris-HCl. To assess if BamA was correctly inserted into the membrane, pelleted membrane fractions were resuspended in 1 ml of PBS containing 5 M urea and mixed continually for 1 h at 4°C. Urea-washed membranes were then isolated by centrifugation for 1 h at 20,800× g and 4°C and the pelleted material was washed three times with PBS and resuspended in 1 ml of PBS. Protein samples were resolved by SDS-PAGE and analyzed using Western blotting [26]. BamA protein was detected using anti-POTRA BamA antiserum, BamB using anti-BamB antiserum, BamC using anti-BamC antiserum, BamD using anti-BamD antiserum and BamE using anti-BamE antiserum, all raised in rabbit [23], [26]. N-terminal 6His tags were detected using anti-6His mouse monoclonal antibodies (Sigma-Aldrich). Blots were developed using the ECL Plus Western Blotting Detection System (GE Healthcare). HA epitopes were detected using anti-HA antiserum (Sigma-Aldrich) from mouse, with secondary goat anti-mouse antibodies conjugated with alkaline phosphatase (Sigma-Aldrich) and the substrate 5-bromo-4-chloro-3-indolyl-β-d-galactopyranoside as detailed in [26].

### 6His BamA pull down experiments

Cultures of BL21(DE3) cells, containing various pET17b/*6hisbamA* constructs were grown to an OD_600_ of ∼0.8 in 100 ml of LB medium with shaking at 37°C without induction. Cells were isolated by centrifugation and pellets resuspended in 2 ml of resuspension buffer (18 mM potassium phosphate buffer (pH 7.3), 320 mM NaCl, 3 mM KCl, and Complete EDTA-free protease inhibitor cocktail tablets (Roche)) containing 0.5% Triton X-100 (v/v), 100 µg ml^−1^ lysozyme and 350 units ml^−1^ Benzonase Nuclease (Sigma-Aldrich). Triton X-100 was included in the resuspension buffer in order to solubilise the membranes. After incubation on ice for 1 h, samples were centrifuged at 20,800× g for 10 minutes at 4°C. 40 µl of 50% nickel-nitrilotriacetic acid (NTA) resin slurry (Qiagen) was added to 1.4 ml of cleared lysate and continually mixed for 4 h at 4°C. NTA resin was isolated by centrifugation at 420× g for 1 minute and washed three times with 1.5 ml of resuspension buffer containing 0.5% Triton X-100 (v/v) and 70 mM imizadole, twice with 1.5 ml of resuspension buffer containing 70 mM imizadole and a final wash with resuspension buffer. Bound proteins were eluted by resuspending resin in 20 µl of Laemmli buffer (Sigma-Aldrich) and boiling for 5 minutes. As some of the 6HisBamA insertion proteins were produced at low levels, the amount of 6HisBamA in each sample was quantified by Western blotting with anti-POTRA BamA antiserum. Protein samples, containing normalised levels of 6HisBamA, were then separated using SDS-PAGE and analysed by Western blotting [26]. This was not possible for the Y141 and L231 insertion constructs, as 6HisBamA containing these insertions bound extremely poorly to the NTA resin.

### Immunofluorescence analysis

Fixation and preparation of bacterial cells for live cell imaging was performed as in [27]. Poly-L-lysine-coated coverslips loaded with fixed cells were washed three times with PBS, and nonspecific binding sites were blocked for 1 h in PBS containing 1% bovine serum albumin (Europa Bioproducts). Coverslips were incubated with 1∶500 anti-HA tag antibody (Sigma-Aldrich) for 1 h, washed three times with PBS, and incubated for an additional 1 h with Alexa Fluor® 488 goat anti-rabbit IgG. The coverslips were then washed three times with PBS, mounted onto glass slides, and visualized using either phase contrast (shown inverted) or fluorescence using Leica DMRE fluorescence microscope (100× objective) -DC200 digital camera system.

### Homology modelling of the full-length BamA protein

To generate the homology-based model of the *E.coli* BamA protein (GenBank AAC73288) we analysed the sequences of over 1500 BamA/Omp85 homologues from a wide range of proteobacteria (α, β, γ, δ and ε), using both iterative-pairwise (M-coffee [28] and MAFFT [29]) and structural (3D-coffee [28] and SAM-T08 [30]) alignment algorithms. The resulting multiple alignments were examined and manually adjusted. Based on these alignments, several models were generated using SAM-T08 [30], RaptorX [31] and I-TASSER [32] using the structure of FhaC (2QDZ) [13] as a structural template and a set of additional distance restraints. Resulting models were superposed, analysed and a composite final model was created and manually refined using Coot [33]. Missing loops were built *de novo*, and the barrel was fused with a composite of POTRA_1_ to POTRA_5_ based on the available structures (3EFC and 3OG5) [8], [10], maximising the overlap with the known position of POTRA_1_ to POTRA_2_ as observed in FhaC structure [13]. Structural alignments were visualised with ESPript [34] and 3D structural models with PyMol [35].

## Results

### Linker scanning mutagenesis of BamA

BamA is an essential protein in *E. coli* and homologues are found in all Gram-negative bacteria [3], [5]. In spite of its importance, no study has systematically mutagenized BamA to determine regions of both structural and functional importance. To address this we carried out linker scanning mutagenesis of *bamA* and examined the ability of each insertion mutant to function in the *E. coli* K-12 BamA depletion strain JWD3 [20]. In JWD3 chromosomally-encoded BamA is only expressed in the presence of arabinose, whilst in its absence, BamA expression is shut down and BamA levels are depleted by successive cell divisions, resulting in the cessation of growth and cell death. Depletion can be rescued by providing a functional plasmid encoded copy of *bamA* (*i.e.* pET17b/*bamA*), which expresses BamA to similar levels seen in JWD3 in the presence of arabinose (Fig. S3). Note that expression of BamA form pET17b/*bamA* is due to leaky expression and is not dependent on T7 RNA polymerase. Random linker scanning mutagenesis of pET17b/*bamA* resulted in the isolation of 87 independent BamA insertion constructs, which were each used to express a version of BamA containing a distinct 5 amino acid insertion (Table S3). The ability of each construct to rescue BamA depletion in JWD3 was then assessed by streaking cells onto agar plates without arabinose. Constructs that rescued depletion were analysed further for their ability to maintain the outer membrane barrier function in the absence of wild-type BamA by streaking cells onto agar plates containing different concentrations of the antibiotic vancomycin (Table S3). *E. coli* K-12 is normally insensitive to high concentrations of vancomycin, however, mutations which disrupt outer membrane biogenesis can lead to defects in the outer membrane, resulting in increased vancomycin permeability and susceptibility [36].

### Analysis of N-terminal linker scanning mutants

**Figure 1:**
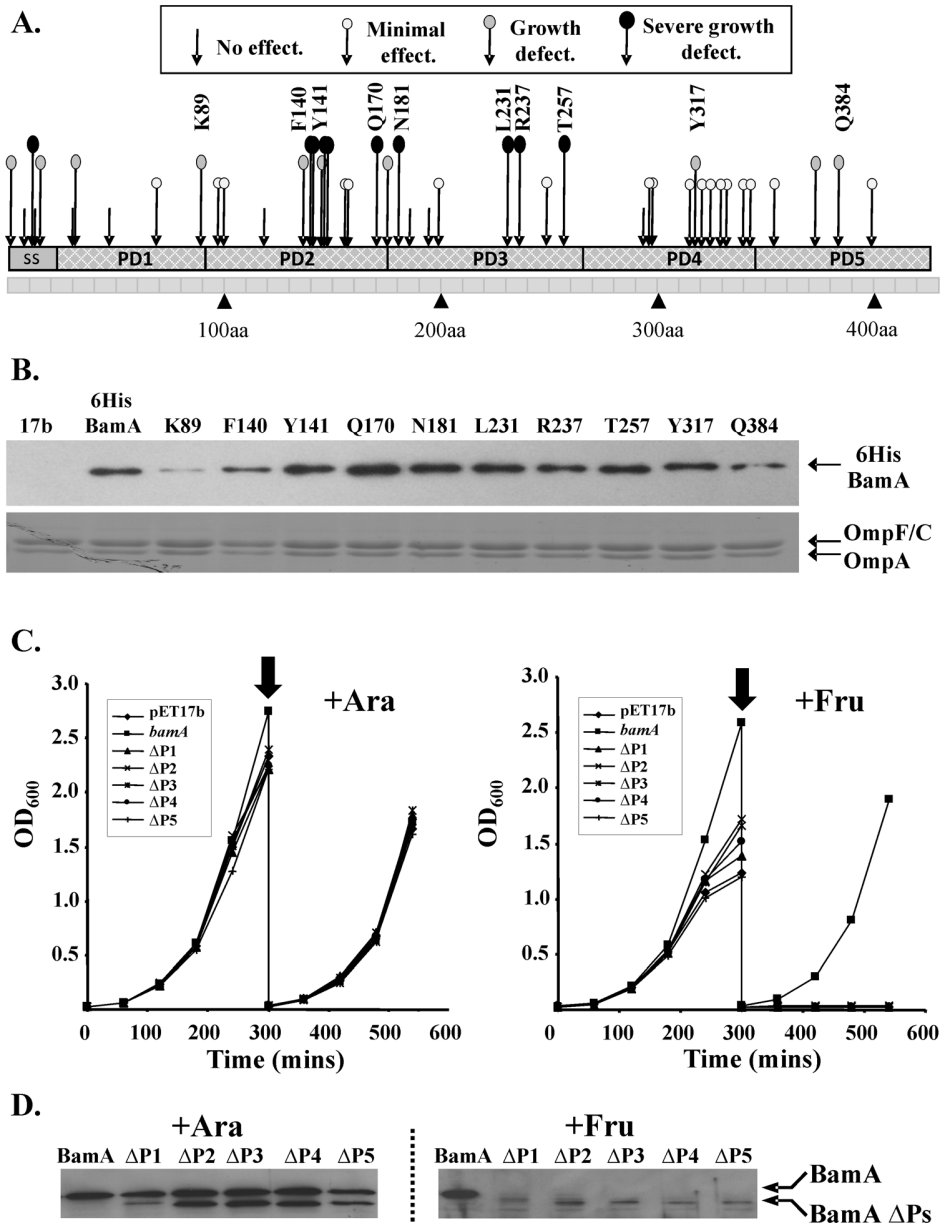
Mutational analysis of the BamA POTRA domains. (A) The panel details the location of 5 amino acid insertions within the BamA POTRA domains. The BamA signal sequence (ss) and POTRA domains (PD1 to PD5) are aligned with the aa sequence number. The function of insertions was monitored by their ability to rescue BamA depletion and/or grow in the presence of vancomycin (*i.e.* 37.5, 75 and 150 µg ml^−1^). A severe growth defect (black lollipops) was defined as either an inability to rescue BamA depletion or to allow growth in the presence of vancomycin. Constructs producing a growth defect (grey lollipops) allowed growth on only 37.5 µg ml^−1^ vancomycin, whilst constructs which grew at 75 µg ml^−1^ vancomycin caused a minimal effect (white lollipops). Constructs which allowed growth at all vancomycin concentrations tested (arrows) had no effect. (B) Detection of BamA POTRA insertions. N-terminal 6His tags were introduced into the K89, F140, Y141, Q170, N181, L231, R237, T257, Y317 and Q384 insertion constructs, cloned into pET17b. Total membranes were prepared from JWD3 cells containing pET17b and the various pET17b/*6hisbamA* constructs, grown in the presence of arabinose. 1.6 µg of membrane protein was Western blotted with anti-6His antiserum (top) and 4 µg was analysed using SDS-PAGE and stained with Coomassie blue (bottom). (C) Deletion analysis of BamA POTRA domains. The panel shows the growth of JWD3 cells carrying pET17b, pET17b/*bamA* and pET17b containing *bamA* constructs with individual POTRA domains deleted (ΔP1 to ΔP5). Cells were grown in Lennox broth in the presence of arabinose or fructose (+Ara or +Fru). After 300 minutes cultures were sampled and subcultured into fresh medium. (D) A Western blot of normalised total cellular protein samples from JWD3 cells after 300 minutes of growth. Blots were probed with anti-BamA POTRA antiserum.

In total 46 insertions were isolated which mapped within the N-terminal region of BamA (Met1 to Arg421), with insertions located within the signal sequence and all 5 POTRA domains (Fig. 1). The effect of insertions within the signal sequence were variable, being relatively severe in some cases and having no effect in others. As this likely reflects the ability of each mutant protein to be targeted to the Sec translocon [1], these insertions were not characterised further. Concerning the POTRA domains, the majority of insertions were located within POTRA_2_, POTRA_3_ and POTRA_4_, with fewer associated with POTRA_1_ and POTRA_5_. Only insertions within POTRA_2_ and POTRA_3_ caused severe defects, with constructs being unable to rescue BamA depletion and/or grow in the presence of any concentrations of vancomycin tested (Table S3). Analysis of the structures of the BamA POTRA domains suggested that some of the insertions with severe effects might disrupt the folding of POTRA domains (*e.g.* insertions F140, Y141, L231 and Q384) or the orientation of POTRA domains with one another (*e.g.* insertions K89 and Q170) (Fig. S4). To confirm that mutant BamA proteins were produced, we generated N-terminal 6His-tagged versions of insertions K89, F140, Y141, Q170, N181, L231, R237, T257, Y317 and Q384, cloned into pET17b, and examined their expression by Western blotting. Note that the 6His versions of each insertion behaved similarly to non-tagged versions in their ability to rescue BamA depletion and in their sensitivity to vancomycin (Table S4). Plasmids expressing each 6His-tagged construct were transformed into JWD3 cells and grown in liquid media under conditions which expressed wild-type BamA (*i.e.* in the presence of arabinose). Total cellular membranes were prepared and samples subjected to Western blotting with anti-6His antiserum. Results in Fig. 1B show that all 6HisBamA insertion proteins were detected, indicating that the proteins were both expressed and associated with the membrane fraction. In most instances, expression levels were similar to those of 6HisBamA, indicating that these insertions did not perturb protein stability. Insertions K89 and F140 were the exception, being produced at lower levels. Thus, their effects could be due to decreased protein expression rather than direct effects of mutating the POTRA domain. Pull down experiments were used to examine whether the BAM lipoproteins were bound to each 6HisBamA insertion. Membranes were solubilised using Triton X-100 detergent and 6HisBamA proteins, with the associated BAM complex members, were purified using NTA resin. Results in Fig. S5 show that 6HisBamA bound specifically to the NTA resin and that BamB, BamC and BamD were still associated with the majority of 6HisBamA proteins tested, indicating that the POTRA domains were correctly folded and that most insertions did not greatly perturb lipoprotein binding. Insertions Y141 and L231 were the exception. As little 6HisBamA protein carrying these insertions bound to the NTA resin, this implies that these proteins were either misfolded or that the N-terminal 6His tag was somehow inaccessible. Interestingly, the Q170 and T257 6HisBamA insertion proteins bound lower levels of BamC and BamD, suggesting that these insertions might interfere with BAM complex assembly.

Previously, Kim *et al.* [6] generated a series of BamA constructs in which the POTRA domains POTRA_1_ to POTRA_4_ were individually deleted. These researchers demonstrated that POTRA_1_ and POTRA_2_ were dispensable for *in vivo* growth. The effect of the POTRA_5_ deletion was not tested due to problems with toxicity [6]. Interestingly, our linker scanning analysis indicated that POTRA_2_ is essential for growth. Therefore, we generated an identical set of BamA POTRA deletions, cloned into pET17b, and examined their ability to rescue BamA depletion in strain JWD3. Cells were grown in liquid medium and supplemented with either arabinose or fructose. Results in Fig. 1C show that none of these POTRA deletion constructs could rescue BamA depletion in the presence of fructose, even though Western blotting of total cellular protein with anti-BamA POTRA antiserum indicated that each protein was expressed (Fig. 1D). Similarly, none of the POTRA deletions could rescue BamA depletion on nutrient or LB agar plates when cells were incubated at 37°C, 30°C or at room temperature, even after 4 days growth (Fig. S6). Very limited growth of JWD3 cells carrying the POTRA_2_ deletion construct did occur on agar plates made with M9 minimal medium in the absence of arabinose, however, this was only evident after 40 h incubation (Fig. S6). Thus, we conclude, that in our BamA rescue-based experimental system, all POTRA domains are essential for normal laboratory growth and viability.

### Analysis of C-terminal linker scanning mutants

**Figure 2:**
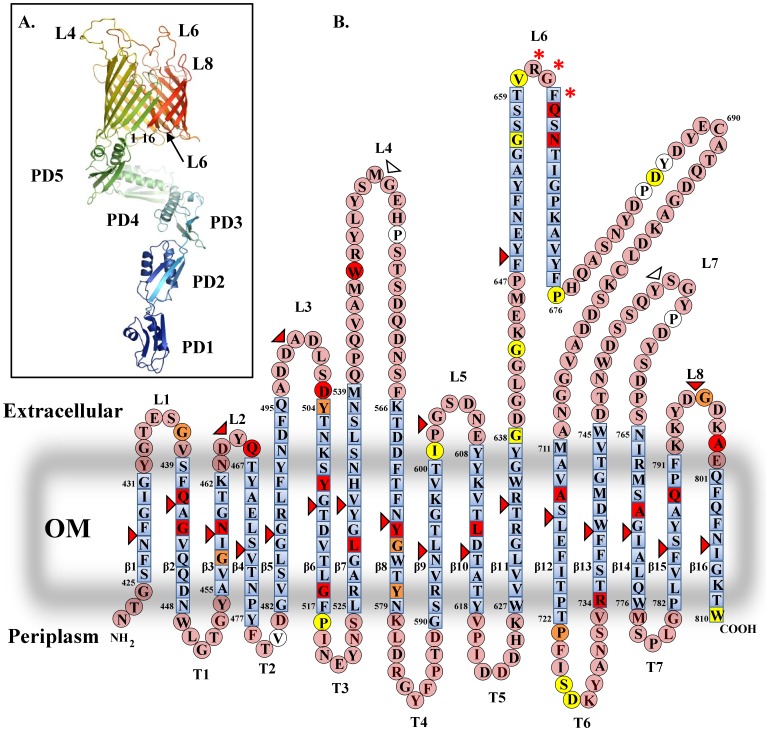
Topology model of the BamA β-barrel. (A) The panel shows a model of BamA, displaying the five POTRA domains and β-barrel of BamA. The model was generated by combining the available crystal structures of BamA POTRA_1_ to POTRA_4_ (3ECF) [8] and POTRA_4_ and POTRA_5_ (3OG5) [10] with the model of the BamA β-barrel generated in this study using Coot [33] and is visualised using PyMol [35]. POTRA domains are indicated (PD1 to PD5), as are β-strands β1 and β16. The tip of loop L6 has been placed within the pore of the β-barrel lumen and has been modelled as a β-hairpin. (B) The panel shows a topology model of the BamA β-barrel (N422 to W810) derived from bioinformatics predictions. Amino acids within β-strand regions are shown as blue squares and those in external loops and periplasmic turns are shown as pink circles. β-strands β1 to β16, extracellular loops L1 to L8 and periplasmic turns T1 to T7 are indicated. The tip region of L6 is predicted to form a β-turn and the RGF motif important in BamA function is starred [14], [15]. The figure also details the position of 5 amino acid insertions isolated by linker scanning mutagenesis (Table S3). Insertions that either failed to rescue BamA depletion or did not allow growth in the presence of vancomycin (severe mutations) are coloured red, whilst insertion constructs which allowed growth on vancomycin concentration of 37.5, 75 and 150 µg ml^−1^ are coloured orange, yellow and white, respectively. The location of HA epitopes inserted within the β-barrel is also indicated using triangles (Table S5). The severity of the insertion is colour coded as for the linker scanning mutations.

In order to predict the boundaries of β-strands, internal turns and external loops of BamA we used homology modelling to generate a model of the BamA β-barrel (Fig. 2). The C-terminal β-barrel domain of BamA was predicted to fold into a 16 stranded β-barrel (strands β1 to β16) with 7 periplasmic turns (T1 to T7) and 8 extracellular loops (L1 to L8). Linker scanning mutagenesis isolated 41 insertions which were located within the β-barrel domain of BamA (Asn422 to Trp810), and targeted all the predicted extracellular loops, most β-strands and relatively few of the periplasmic turns (Fig. 2). We hypothesised that disruption of β-strands would compromise barrel folding and BamA function. Depletion experiments and vancomycin growth assays using strain JWD3 indicated that insertions within β-strands generally caused severe phenotypic effects. These results are consistent with our model (Fig. 2 and Table S3). Insertions within loops L4, L6 and L8 also had severe effects, suggesting that these external loops may play a role in OMP biogenesis. Other insertions on the boundary between predicted loops and β-strands (*e.g.* Q466 and D503) were also severe in nature and possibly suggest that loops L2 and L3 are also important. However, we note that some insertions within loops L1, L4, L6, L7 and L8 were less severe or completely tolerated, indicating that many loops can accommodate insertions and that the site of insertion is important for the phenotype observed. As the number of insertions within turns was limited, we are not able to ascertain whether most turns are important. However, as T6 was able to tolerate a number of insertions it is likely that this turn is not essential (Fig. 2).

**Figure 3:**
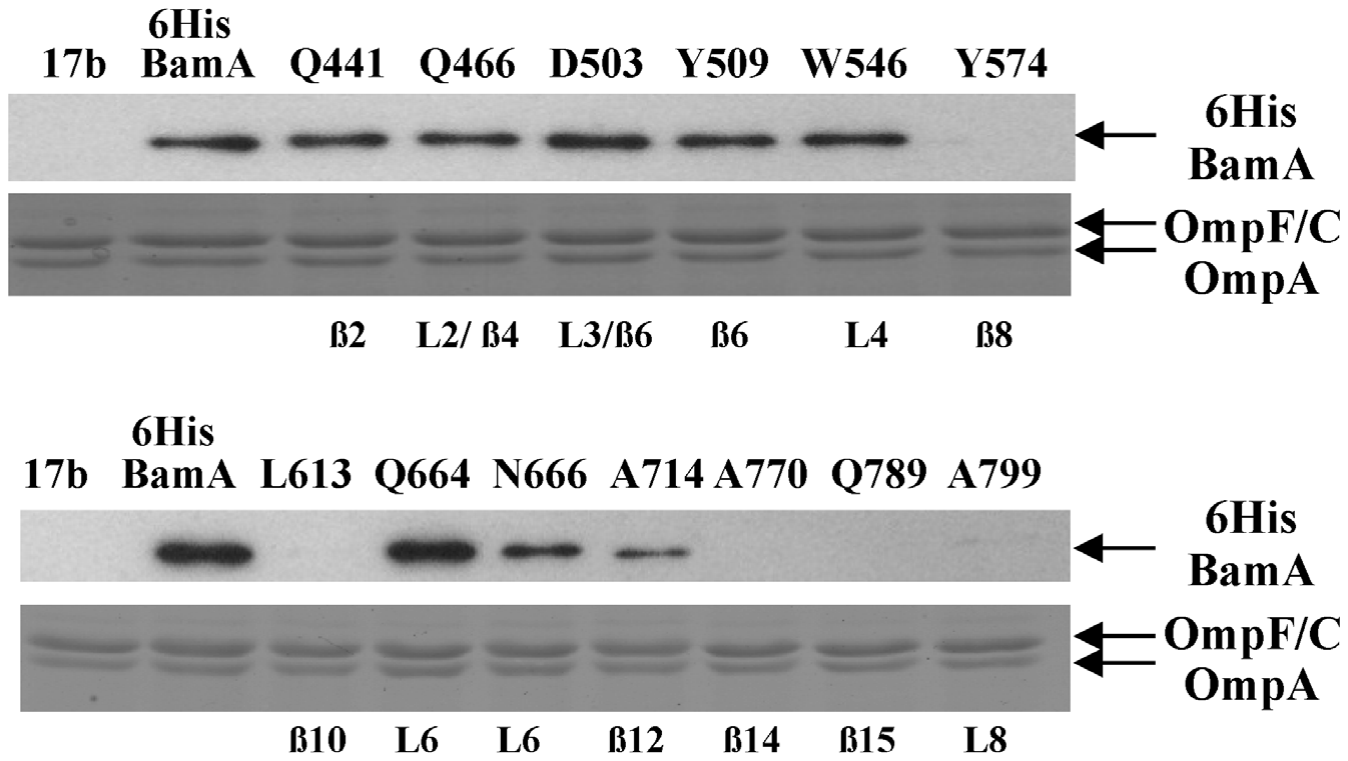
Mutational Analysis of the BamA β-barrel. Detection of BamA proteins carrying insertions within the β-barrel domain. N-terminal 6His tags were introduced into the Q441, Q466, D503, Y509, W546, Y574, L613, Q664, N666, A714, A770, Q789 and A799 insertion constructs cloned into pET17b (see Fig. 2). Total membranes were prepared from JWD3 cells, containing pET17b, pET17b/*6hisbamA* or pET17b carrying 6His insertion mutants, grown in the presence of arabinose. 1.6 µg of total membrane protein was subjected to Western blotting with anti-6His antiserum (top panel) and 4 µg of protein was analysed using SDS-PAGE and stained with Coomassie blue (bottom panel). The location of 6HisBamA proteins, OmpF, OmpC and OmpA are indicated. The location of each insertion with respect to the secondary structure detailed in Fig. 2 is also given.

To examine whether BamA proteins carrying C-terminal insertions could be detected, we generated N-terminal 6His-tagged versions of 13 insertions (*i.e.* Q441, Q466, D503, Y509, W546, Y574, L613, Q664, N666, A714, A770, Q789 and A799) cloned into pET17b. None of the 6His versions rescued BamA depletion and all behaved similarly to the non-tagged versions (Table S4). Total cellular membranes were prepared from JWD3 cells which carried each insertion construct and had been grown in the presence of arabinose. The membrane samples were subjected to Western blotting with anti-6His antiserum. Results shown in Fig. 3 reveal that many of the BamA proteins carrying insertions within predicted β-strands were not detected (*i.e.* β8 (Y574), β10 (L613), β14 (A770) and β15 (Q789)) or produced at much lower levels than wild-type 6HisBamA (*i.e.* β12 (A714)). This suggests that insertion into these regions destabilizes the BamA β-barrel and is consistent with our topology model. Some insertions within proposed strands were detected (*i.e.* β2 (Q441) and β6 (Y509)) suggesting that although these insertions were unable to rescue BamA depletion, the nature of the insertion did not lead to protein degradation. BamA proteins containing insertions within loops L2 (Q466), L3 (D503), L4 (W546) and L6 (Q664 and N666) were also detected, whilst the insertion within L8 (A799) was not. Thus, we can conclude that loops L2, L3, L4 and L6 are likely important.

It has been proposed that the conformation of the L6 loop within BamA is controlled by the associated BAM lipoproteins, raising the possibility that a direct interaction occurs between BamD/BamE and L6 [16], [37]. Therefore, we examined the association of BamB, BamC and BamD with 6HisBamA proteins carrying insertions within L6 (*i.e.* Q664 and N666). Pull down experiments show that BAM lipoproteins were still associated with both proteins (Fig. S5), indicating that these insertions did not interfere with lipoprotein binding.

### Analysis of C-terminal HA insertion constructs

**Figure 4:**
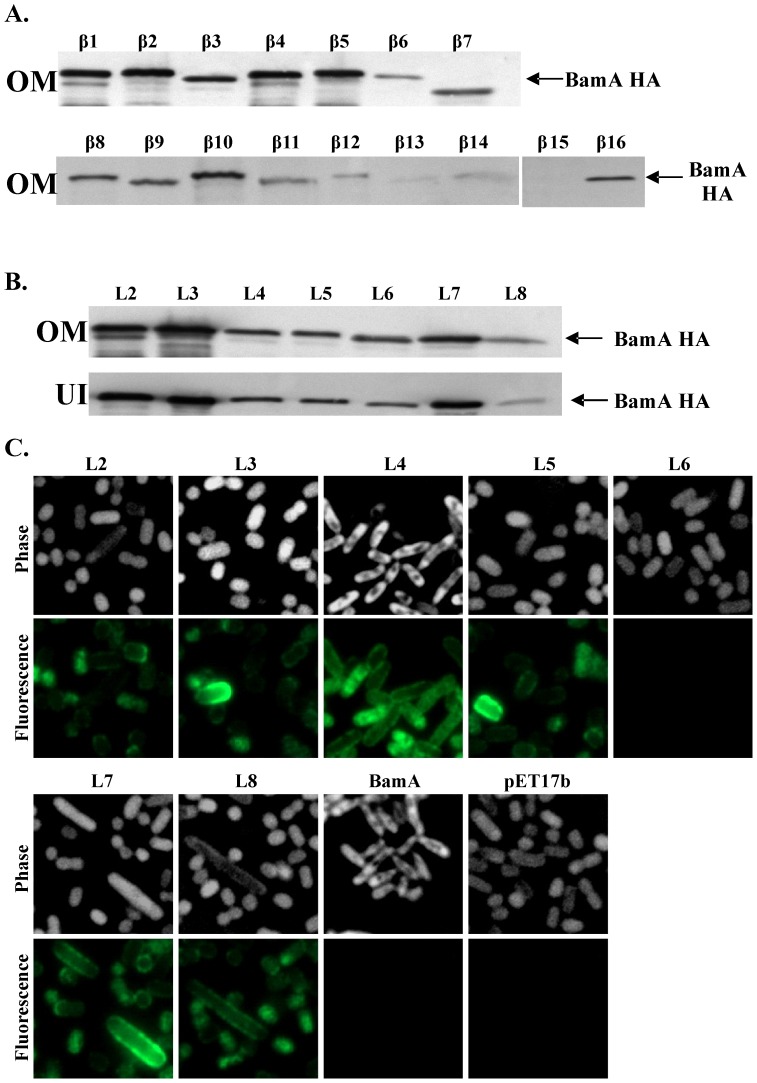
Analysis of HA epitopes within the BamA β-barrel. The figure shows the detection of BamA proteins carrying HA insertions within the β-strands and external loops of the BamA β-barrel. HA epitopes were introduced into β-strands (β1 to β16) and loops (L2 to L8) (see Fig. 2) and *bamA* insertion constructs were cloned into pET17b. Outer membranes (OM) were prepared from JWD3 cells containing each construct and normalised protein samples were subjected to Western blotting with anti-HA antiserum. (A) shows a Western blot analysis of BamA proteins carrying HA epitopes in β-strands β1 to β16 and (B), in loops L2 to L8. Panel (B) also shows the urea insoluble fraction (UI) obtained after outer membrane preparations were washed with urea. All BamA proteins carrying HA epitopes in their loop domains were localised within the urea insoluble fraction, indicating that they are correctly folded within the membrane. (C) Immunofluorescence analysis of BamA constructs carrying HA epitopes within external loops. JWD3 cells, containing pET17b, pET17b/*bamA* or pET17b carrying HA insertions in L2 to L8, were fixed, probed with anti-HA and Alexa Fluor® 488 antibody and visualized using phase contrast (shown inverted) and fluorescence microscopy.

To probe our model further we introduced the DNA encoding a 9 amino acid HA epitope (encoding residues YPYDVPDYA) into the regions of the BamA β-barrel predicted to form β-strands and external loops (Fig. 2 and Table S5). We reasoned that insertion of HA epitopes would disrupt strands, severely affecting BamA folding and function, whilst insertions into loops might be tolerated, provided they did not affect functionally important regions. Western blotting of outer membrane preparations from JWD3 cells carrying BamA constructs with β-strand localized HA epitopes indicated that most insertion proteins were expressed (Fig. 4A). However, some of the HA-containing BamA proteins were smaller in size and expressed to lower levels, suggesting that they may be unstable and degraded. Consistent with our modeling predictions, insertions within β-strands resulted in BamA constructs which were drastically compromised for their ability to rescue BamA depletion (Fig. 2 and Table S5). Similarly, HA epitope insertions within loops L2, L3, L5, L6 and L8 were severe in nature, suggesting that these loops might be important. Insertions within loops L4 and L7 were tolerated, having no appreciable effect on cell viability or outer membrane integrity (Table S5). Note we were unable to isolate an insertion within L1. Western blotting of outer membrane fractions from JWD3 cells carrying each HA epitope loop insertion indicated that all constructs were expressed (Fig. 4B). Furthermore, washing membrane preparations with 5 M urea indicated that all loop insertions were correctly folded, being present in the urea insoluble fraction (Fig. 4B).

The insertion of HA epitopes into the loops of BamA also enabled us to probe whether HA tags were surface exposed using immunofluorescence analysis. Whole JWD3 cells, carrying plasmids which expressed each BamA loop HA construct, were fixed and probed with anti-HA antiserum, before being subjected to phase contrast and fluorescence microscopy. Results in Fig. 4C show that HA insertions in L2, L3, L4, L5, L7 and L8 were detected, indicating that these epitopes were surface localized. This is consistent with our prediction that these elements of BamA form exposed external loops (Fig. 2). Surprisingly, the HA insertion within loop L6 was not detected. As it has been suggested that L6 is located within the BamA β-barrel [16] it is possible that this part of L6 is not accessible to antibody.

### Analysis of C-terminal loop deletions

**Figure 5:**
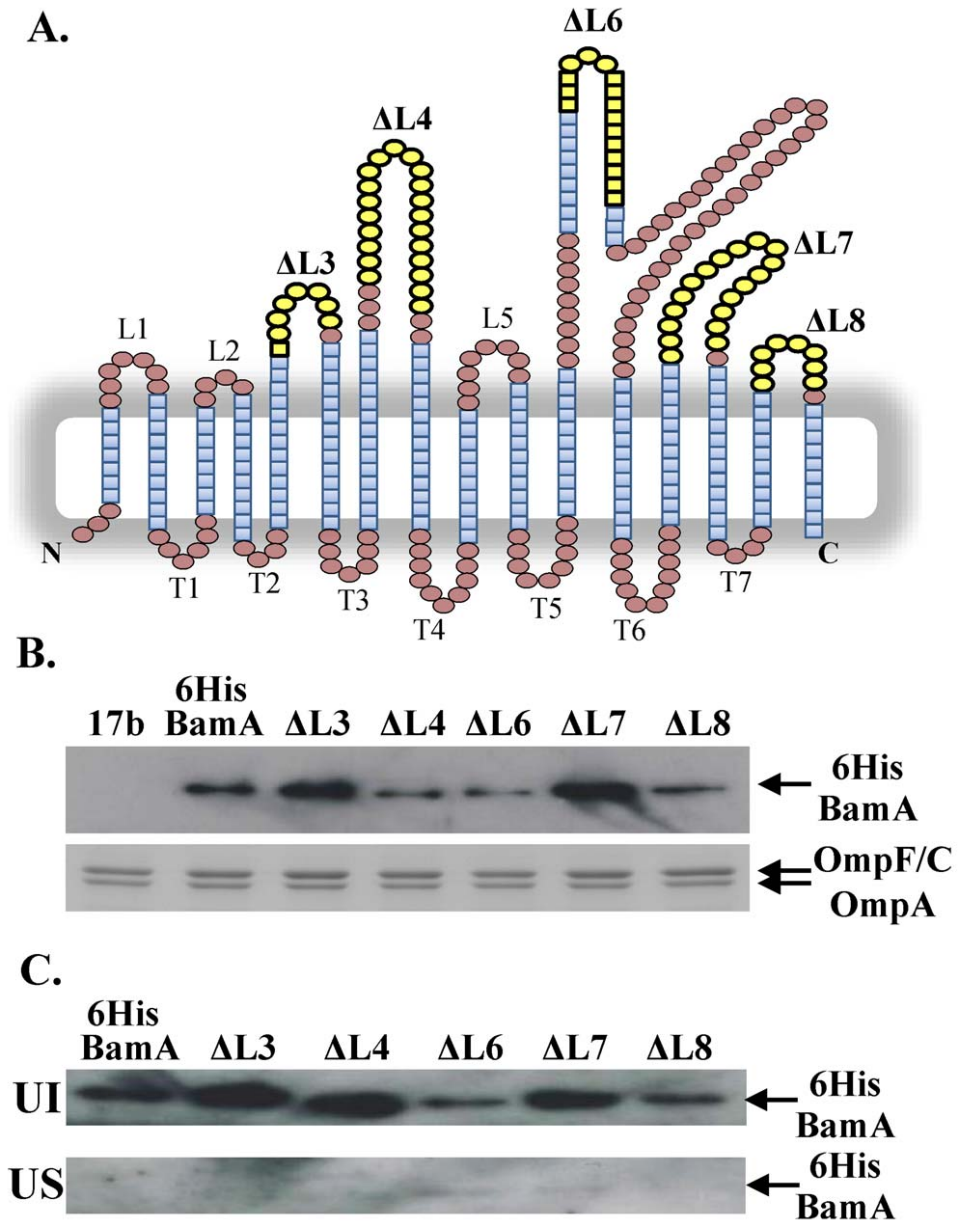
Deletion of BamA extracellular loops. (A) The location of external loop deletions within the BamA β-barrel. The panel shows the location of loop deletions (yellow) introduced into BamA based on the homology model detailed in Fig. 2. For deletions ΔL3, ΔL4, ΔL7 and ΔL8 the deleted sequence was replaced by three glycine residues to maintain flexibility between β-strands. (B) Detection of BamA proteins carrying loop deletions. Total cellular membranes were prepared from JWD3 cells carrying pET17b, pET17b/*6hisbamA* and pET17b carrying 6HisBamA loop deletion constructs (see panel A). 1.6 µg of total membrane protein was subjected to Western blotting with anti-6His antiserum (top panel) and 4 µg of protein was analysed using SDS-PAGE and strained with Coomassie blue (bottom panel). The location of 6HisBamA proteins, OmpF, OmpC and OmpA are indicated. (C) Urea washing of membrane-localised BamA proteins containing loop deletions. Total cellular membranes from JWD3 cells carrying pET17b/*6hisbamA* or pET17b containing 6HisBamA loop deletion constructs were washed with urea and the insoluble (UI) and soluble (US) fractions subjected to Western blotting with anti-6His antiserum. All 6HisBamA proteins were localised within the urea insoluble fraction indicting that they are full folded within the membrane.

Our insertion analysis of the BamA β-barrel indicated that surface loops, other than L6, are likely important for BamA function. To investigate this further we examined the effect of deleting specific loop domains. We were able to isolate 6HisBamA constructs which carried complete deletions of L3, L4, L7 and L8 and a partial deletion of L6, which removes the conserved RGF motif (Fig. 5A). Results in Table S6 show that deletion of either L6 or L4 resulted in non-functional proteins unable to rescue depletion, whilst the removal of either L7 or L8 resulted in severe defects in membrane integrity. However, deletion of L3 was tolerated, with this construct only producing minor defects in membrane permeability. Western blotting of total membrane fractions from JWD3 cells expressing each construct with anti-6His antiserum indicated that all BamA loop deletion proteins were expressed (Fig. 5B). Urea extraction of membranes also indicated that each protein was correctly folded being present in the urea insoluble fraction (Fig. 5C). Thus, we can conclude that L4, L6, L7 and L8 are important.

## Discussion

Bacterial OMPs, which adopt a β-barrel conformation, are diverse in both structure and function, being involved in many different cellular processes such as solute transport, adhesion and toxin delivery [38]–[40]. The BAM complex is central to OMP biogenesis and bacterial viability. Therefore, understanding this sophisticated machine is essential for developing new strategies for targeting and controlling Gram-negative pathogens. To gain insight into the workings of the BAM complex we have used genetic, biochemical and modeling approaches to identify important functional and structural regions of the *E. coli* BamA protein and have developed a topological prediction of the BamA β-barrel.

Our linker scanning mutagenesis isolated a number of insertions in both the POTRA and β-barrel domains of BamA, which led to severe phenotypic defects and indicate that both domains play a role in OMP biogenesis. Interestingly, all severe insertions within the POTRA domains were associated with POTRA_2_ and POTRA_3_, whilst none were within POTRA_1_, POTRA_4_ and POTRA_5_, even though these POTRA domains are essential (Fig. 1) [6]. Due to the methodology of library construction (see Materials and Methods) it is unlikely that the failure to isolate such mutations was due to toxicity but rather reflect that our BamA mutagenesis had not reached saturation. Our observation that all POTRA domains are essential is in contrast to those of Kim *et al.* [6], who initially showed that POTRA_1_ and POTRA_2_ were dispensable for BamA function, although cells bearing these deletions grew extremely poorly. Our POTRA deletions were identical to those used in that study (Table S1 and [6]) and were expressed (Fig. 1). In our experiments, recombinant BamA expression was reliant on leaky low level expression from pET17b, which led to similar levels of BamA to that observed when the depletion strain JWD3 was grown in the presence of arabinose (Fig. S3). The constructs used by Kim *et al*. [6] were cloned into expression vector pZS21 in which expression was constitutive from the deregulated P_LTetO-1_ promoter [6]. As expression levels of BamA are important [41], we believe that this, coupled with any differences in the depletion strains used could account for the observed differences. Thus, in our experimental system all BamA POTRAs are required for normal growth. Interestingly, in the *Neisseria meningitidis* BamA homologue, POTRA_1_ to POTRA_4_ can be deleted without effecting cell viability, indicating that there are differences between organisms [42].

In order to rationalise our mutagenesis of BamA, we generated a homology model of the BamA β-barrel. We predict that, as for FhaC, the C-terminal domain of BamA folds into a 16 stranded β-barrel with eight extracellular loops and that L6 is particularly extended. Based on homology with FhaC we generated a 3D model of BamA, placing L6 within the β-barrel and suggest that L6 forms a β-hairpin with the conserved RGF motif at its tip (Fig. 2). As expected, linker scanning and HA epitope insertions within predicted transmembrane β-strands resulted in severe BamA phenotypes. This is consistent with our predictions that these regions adopt a β-strand conformation that spans the outer membrane (Fig. 2). The HA epitope insertions within loops and the deletion of loops themselves had varying effects, depending on the particular loop modified. However, in each case the engineered BamA protein was expressed and correctly folded within the membrane, as judged by urea extraction. This indicates that the manipulation of each predicted loop did not greatly perturb the folding of the barrel and supports our predictions that these regions form extracellular loops. As would be expected for extracellular loops, we were also able to show that loop-associated HA tags were surface-localised, with the obvious exception of the HA L6 insertion. Thus, based on genetic and functional testing of our model, we propose a consensus topology for the BamA β-barrel.

Our model of the BamA β-barrel has relied heavily on the secondary structure adopted by FhaC [13]. Alignment of the BamA and FhaC β-barrels indicate that the two sequences have direct correspondence for the majority of their alignment, which, however, breaks down at two positions (Fig. S7). The first of these corresponds to positions G590 to I601 in BamA (*i.e.* β9) and appears to be an insertion within BamA. However, our analysis of the secondary structure predictions and sequence conservation reveals a rather more intriguing interpretation. Based on the analysis of the sequence conservation within the BamA family (analysis of >1500 homologues), we predict that from Q540 there is a mismatch in the secondary structure of BamA with that of FhaC. Topologically BamA and FhaC are closely matched from β1 to β7 inclusive, which is supported by the remarkable conservation of sequence and the length of the β-strands. However, the sequence of β8 in FhaC is predicted to correspond to the extended loop L4 in BamA. Furthermore, in BamA, the region K566 to N579 corresponds to the inward pointing β8, whilst the equivalent sequence in FhaC forms the outward pointing β9 (see Fig. S8). The BamA β9 has no equivalence in the FhaC sequence and appears as an insertion in the alignment (Fig. S7). After this point, the β10 strands in both BamA and FhaC are in topologically equivalent positions. From an evolutionary point of view it is more likely that the original β9 in the BamA-like precursor of FhaC was lost and this resulted in an adaptation of loop L4 to form β8, maintaining sequence conservation but greatly shortening the FhaC L4 and rearranging membrane topology. The fact that the region G590 to I601 is indeed β9 in BamA is supported by the high level of sequence conservation and lack of length variability of this region (Fig. S9), as well as the severe phenotype of the β9 HA insertion within this region (Fig. 2). This is in contrast to the predicted L4 region, which is highly divergent both in sequence and length within the BamA family (Fig. S9). Our assignment of this region as a loop is further supported by the complete tolerance of the L4 HA epitope insertion at M552 and its surface localisation (Figs. 2 and 4C). In addition to this, a second region of significant dissimilarity in the BamA/FhaC alignment is observed within loop L6 and at the L6/β12 junction, with the position of β12 in FhaC is shifted in comparison to BamA (Fig. S7). Alignment of BamA orthologues (Fig. S9) places the β12 of BamA between M711 and T722, which is consistent with the effects observed for the β12 HA epitope insertion and for the linker scanning insertions A714, S726 and D727 (Fig. 2). Thus, in BamA loop L6 is more extended and it seems that the positioning of the L6/β12 junction differs to FhaC. Why FhaC and BamA appear to have slightly different topological organisations in these two regions is unclear, though this may reflect the different substrates that each of these proteins interact with. FhaC is responsible for secreting the FhaC substrate, FHA, across the outer membrane in *B. pertussis*, whilst BamA inserts many different OMPs into a lipid environment.

A major finding of this study is that many of the extracellular loops are also important. Linker scanning insertions within L2 (Q466), L3 (D503), L4 (W546) and L6 (Q664 and N666) resulted in severe BamA phenotypes, whilst an insertion in L8 (A799) greatly affected BamA stability (Figs. 2 and 3). HA epitope insertions into L2, L3, L5, L6 and L8 also produced severe effects (Fig. 2). Deletion of L4 or part of L6 produced folded proteins which were unable to rescue BamA depletion, whilst removal of L7 and L8 resulted in outer membrane permeability defects (Table S6). Thus, it is clear that the surface-exposed loops of BamA greatly influence the folding events which take place on the periplasmic face of the outer membrane.

The role that L6 plays in OMP biogenesis has recently received much attention due to the homology that BamA displays with the FhaC two-partner secretion system protein. In the crystal structure of FhaC, L6 is located within the FhaC β-barrel and this long loop is essential for secretion of FHA [13], [14], [43]. It has been proposed that L6 is similarly located within the BamA β-barrel [16] and alignments of BamA orthologues indicate that the proposed tip of L6 (M646 to A672), encompassing the RGF motif, is conserved (Fig. S9) [14] and is essential for BamA function [15]. Consistent with this, deletion of this region and linker scanning insertions directly after the RGF motif (Q664 and N666) resulted in severe BamA phenotypes. Surprisingly, most linker scanning insertions within L6 were tolerated, having only minor effects on membrane integrity (Fig. 2). From the alignment of BamA orthologues it is clear that L6 is smaller in other organisms (*e.g. Neisseria gonorrhoeae*) (Fig. S9) and consequently L6 sequences after position A672 in *E. coli* BamA may be non-essential. Our HA epitope insertion within L6 at position F648 resulted in a protein which was unable to rescue BamA depletion but that was stably inserted into the outer membrane. In spite of this, we were unable to detect a surface-localised HA tag using immunofluorescence, indicating that this region of L6 is inaccessible to antibody. Our 3D model of BamA suggests that this region of L6 is located within the lumen of the BamA β-barrel (Fig. 2). Similar issues with accessibility have been documented for the two cysteine residues within L6 (*i.e.* C690 and C700) [16]. Thus, the simplest explanation is that L6 is occluded within the barrel pore in a similar manner to L6 for FhaC [13], [16]. It has been suggested that the BamA β-barrel and L6 both undergo conformational changes during OMP folding and that L6 becomes surface exposed [16]. These transitions are thought to be driven by the BamD and BamE lipoproteins. Interestingly, the insertions at the tip of L6 (Q664 and N666) did not prevent the binding of the BAM lipoproteins to BamA, suggesting that if a L6/BamD/BamE interaction occurs, then the major stabilizing interaction between BamA and BamDCE is still via the POTRA domains.

A novel finding of this study is that external loops, other than L6, are important. Here, we have identified in all loop domains insertions and/or deletions that result in severe BamA phenotypes. The exception to this is L1, which is predicted to be a small loop that is well conserved between BamA orthologues (Fig. S9). A linker scanning insertion in L1 (G347) caused a defect in membrane integrity (Fig. 2), however, we were unable to isolate a stable L1 HA epitope insertion. It is unclear whether this is of significance and at present we are unable to confirm whether L1 is functionally important. Loops L2, L5 and L8 are well conserved between BamA orthologues, which likely suggests that they are important functionally and/or structurally (Fig. S9). Loops L4, L3 and L7 are less conserved between orthologues and more variable in length, perhaps suggesting a minor role. However, we note that there is a conserved motif in L4 (548 YLYS 551) and that the nearby insertion at W546 resulted in a severe BamA phenotype (Fig. S9 and Fig. 2), suggesting that L4 is of importance. Interestingly, in the case of L3, deletion of the entire loop demonstrated that L3 is dispensable, whilst insertions within the loop suggested the contrary. The simplest explanation for this observation is that insertions within L3 have altered the loop's conformation and this sterically hinders BamA, preventing it from functioning.

It is clear that, in addition to the POTRA domains, the BamA β-barrel is integrally involved in OMP biogenesis and its contribution is greater than simply tethering BamA to the outer membrane. The importance of L6 has been demonstrated by this study and others [15], [16] and it is now evident that other external loops are critically important. It has been suggested that both the BamA β-barrel and L6 undergo conformational changes during OMP folding [16] and it is conceivable that extracellular loops could aid these movements or stabilise the different conformations that the barrel adopts. Interestingly, many of the extracellular loop domains are conspicuously charged, which could be important for BamA function.

During the reviewing of this manuscript the crystal structures of the BamA homologues from *N. gonorrhoeae* and *H. ducreyi* were reported, revealing that the C-terminal domain of each protein adopts a 16-stranded β-barrel [17]. Importantly, our proposed topology for the *E. coli* BamA β-barrel is in excellent agreement with that presented by Noinaj *et al.* [17]. Many of our C-terminal linker scanning and HA insertion mutants, which we predicted are located within β-strands, were either not expressed or produced at low levels (Figs. 3 and 4A). This new data confirms our predictions and it is likely that these insertions destabilize the BamA β-barrel, leading to protein degradation. In both BamA structures, the loop domains form a stabilizing dome over the top of the β-barrel and the L4/β8 and L6/β12 boundaries are as we have predicted for BamA orthologues [17]. Interestingly, the evolutionary conserved YLYS motif, identified in L4 using bioinformatics and insertion mutagenesis (insertion W546) in our study, was shown to form a surface-exposed α-helix within L4 [17]. As expected, L6 was partially located within the barrel lumen and deletion of non-conserved L6 residues P676 to C700 was shown to be tolerated [17]. Surprisingly, the L6 RGF motif interacted with conserved residues in β12 and β13 of the barrel (E717 and D740 in *E. coli* BamA). These interactions appear to be important in stabilizing the *E. coli* BamA β-barrel [17] and it is noteworthy that β12 and β13 HA insertions and the linker scanning insertion at A714 are produced at low levels, suggesting they may promote barrel instability (Figs. 3 and 4A). Based on the crystal structures and modelling simulations Noinaj *et al*.[17] proposed that during OMP biogenesis BamA β-strands β1 and β16 separate and act as templates for folding OMPs. Our study isolated HA epitope insertions within both of these strands and although outer membrane localised proteins were detected (Fig. 4A), neither insertion rescued BamA depletion. At present it is unclear how our loop domain mutations influence BamA. As it has been proposed that by forming a dome over the barrel that the loops stabilise the C-terminal domain [17], it is possible that insertions and deletions within loops could destabilise the β-barrel or affect the possible alternative conformations that BamA may adopt. Thus, it is hoped that as the topology of *E. coli* BamA has been finally deciphered more targeted mutational studies will reveal the intricacies of this complex molecular machine and unravel the mechanism by which the BAM complex folds and inserts OMPs into the outer membrane.

## Supporting Information

Table S1 Strains and plasmids used in this work.
(PDF)

Table S2 DNA Primers used in this work.
(PDF)

Table S3 Linker scanning insertions in BamA.
(PDF)

Table S4 6His tagged BamA insertions.
(PDF)

Table S5 HA insertions in BamA.
(PDF)

Table S6 6His tagged BamA barrel loop deletions.
(PDF)

Figure S1 DNA sequence of *bamA*.
(PDF)

Figure S2 Alignment of the BamA sequences.
(PDF)

Figure S3 Depletion of BamA in *E. coli* JWD3 cells.
(PDF)

Figure S4 Location of 5 amino acid insertions within the POTRA domains of BamA.
(PDF)

Figure S5 Pull down experiments with 6HisBamA proteins carrying 5 amino acid insertions.
(PDF)

Figure S6 Depletion analysis of BamA POTRA domains on solid media.
(PDF)

Figure S7 Alignment of the β-barrel domains from BamA and FhaC.
(PDF)

Figure S8 Comparison of the BamA and FhaC secondary structure surrounding loop L4 of the β-barrel domain.
(PDF)

Figure S9 Alignment of BamA/Omp85 orthologues.
(PDF)
