# Supplementary material for: Mutational and Topological Analysis of the Escherichia coli BamA Protein
Source: PLoS One. 2013 Dec 23;8(12):e84512. doi: 10.1371/journal.pone.0084512 (PMC3871556; doi:10.1371/journal.pone.0084512)
Supplement: Table S1 — Strains and plasmids used in this work. (PDF) [file pone.0084512.s001.pdf]

Table S1. Strains and plasmids used in this work.

Bacterial strains.	Relevant genotype.	Reference.
RLG221	*E. coli* K-12 cloning strain. *recA56 araD139 (are-leu)7697 laxX74 glaU galK hsdR strA*	R. Gourse
JWD3	*E. coli* K-12 BamA depletion strain.	[19]
BL21(DE3)	*fhuA2 [lon] ompT gal (λ DE3)[dcm] ΔhsdS*	New England BioLabs
**Bacterial plasmids.**		
pET17b	T7 expression vector (Amp^R)	Novagen
pET17b/ *bamA*	pET17b plasmid which expresses a codon-optimised version of *E. coli* BamA.	This Work
pET17b/ *6hisbamA*	pET17b plasmid which expresses a 6His tagged codon-optimised version of *E. coli* BamA. HHHHHHAA was inserted between A21 and E22.	This Work
pET17b/ *bamA* ΔP1	BamA $POTRA_1$ deletion. Amino acids E22 to K89 deleted and an A inserted between A21 and E90.	This Work
pET17b/ *bamA* ΔP2	BamA $POTRA_2$ deletion. Amino acids P92 to G172 deleted.	This Work
pET17b/ *bamA* ΔP3	BamA $POTRA_3$ deletion. Amino acids V173 to D264 deleted and an A inserted between G172 and Q265.	This Work
pET17b/ *bamA* ΔP4	BamA $POTRA_4$ deletion. Amino acids D264 to N345 deleted and an A inserted between G263 and R346.	This Work
pET17b/ *bamA* ΔP5	BamA $POTRA_5$ deletion. Amino acids N345 to R421 deleted.	This Work
pET17b/ *bamA* L2HA	BamA L2 HA epitope insertion. YPYDVPDYA was inserted between D464 and Y465.	This Work
pET17b/ *bamA* L3HA	BamA L3 HA epitope insertion. YPYDVPDYA was inserted between D498 and A499.	This Work
pET17b/ *bamA* L4HA	BamA L4 HA epitope insertion. YPYDVPDYA was inserted between M552 and G553.	This Work
pET17b/ *bamA* L5HA	BamA L5 HA epitope insertion. YPYDVPDYA was inserted between P602 and G603.	This Work
pET17b/ *bamA* L6HA	BamA L6 HA epitope insertion. YPYDVPDYA was inserted between F648 and Y649.	This Work
pET17b/ *bamA* L7HA	BamA L7 HA epitope insertion. YPYDVPDYA was inserted between Y754 and S755.	This Work
pET17b/ *bamA* L8HA	BamA L8 HA epitope insertion. YPYDVPDYA was inserted between D795 and G796.	This Work
pET17b/ *bamA* β1HA	BamA β1 HA epitope insertion. YPYDVPDYA was inserted between N427 and F428.	This Work
pET17b/ *bamA* β2HA	BamA β2 HA epitope insertion. YPYDVPDYA was inserted between Q441 and A442.	This Work
pET17b/ *bamA* β3HA	BamA β3 HA epitope insertion. YPYDVPDYA was inserted between I458 and N459.	This Work
pET17b/ *bamA* β4HA	BamA β4 HA epitope insertion. YPYDVPDYA was inserted between S472 and V473.	This Work

pET17b/ *bamA* β5HA	BamA β5 HA epitope insertion. YPYDVPDYA was inserted between G486 and G487.	This Work
pET17b/ *bamA* β6HA	BamA β6 HA epitope insertion. YPYDVPDYA was inserted between G510 and T511.	This Work
pET17b/ *bamA* β7HA	BamA β7 HA epitope insertion. YPYDVPDYA was inserted between Y531 and V532.	This Work
pET17b/ *bamA* β8HA	BamA β8 HA epitope insertion. YPYDVPDYA was inserted between N573 and Y574.	This Work
pET17b/ *bamA* β9HA	BamA β9 HA epitope insertion. YPYDVPDYA was inserted between N594 and L595.	This Work
pET17b/ *bamA* β10HA	BamA β10 HA epitope insertion. YPYDVPDYA was inserted between D614 and T615.	This Work
pET17b/ *bamA* β11HA	BamA β11 HA epitope insertion. YPYDVPDYA was inserted between T633 and R634.	This Work
pET17b/ *bamA* β12HA	BamA β12 HA epitope insertion. YPYDVPDYA was inserted between S715 and L716.	This Work
pET17b/ *bamA* β13HA	BamA β13 HA epitope insertion. YPYDVPDYA was inserted between F738 and W739.	This Work
pET17b/ *bamA* β14HA	BamA β14 HA epitope insertion. YPYDVPDYA was inserted between G771 and I772.	This Work
pET17b/ *bamA* β15HA	BamA β15 HA epitope insertion. YPYDVPDYA was inserted between F785 and S786.	This Work
pET17b/ *bamA* β16HA	BamA β16 HA epitope insertion. YPYDVPDYA was inserted between N805 and I806.	This Work
pET17b/ *6hisbamA* ΔL3	6His tagged BamA L3 deletion. Amino acids Q495 to S502 were deleted and replaced with GGG. HHHHHHAA was inserted between A21 and E22.	This Work
pET17b/ *6hisbamA* ΔL4	6His tagged BamA L4 deletion. Amino acids V543 to N563 were deleted and replaced with GGG. HHHHHHAA was inserted between A21 and E22.	This Work
pET17b/ *6hisbamA* ΔL6	6His tagged BamA partial L6 deletion. Amino acids S657 to A672 were deleted. HHHHHHAA was inserted between A21 and E22.	This Work
pET17b/ *6hisbamA* ΔL7	6His tagged BamA L7 deletion. Amino acids D746 to P763 were deleted and replaced with GGG. HHHHHHAA was inserted between A21 and E22.	This Work
pET17b/ *6hisbamA* ΔL8	6His tagged BamA L8 deletion. Amino acids K792 to A799 were deleted and replaced with GGG. HHHHHHAA was inserted between A21 and E22.	This Work
