# Supplementary material for: Mutational and Topological Analysis of the Escherichia coli BamA Protein
Source: PLoS One. 2013 Dec 23;8(12):e84512. doi: 10.1371/journal.pone.0084512 (PMC3871556; doi:10.1371/journal.pone.0084512)
Supplement: Table S2 — DNA Primers used in this work. (PDF) [file pone.0084512.s002.pdf]

Table S2. DNA Primers used in this work. Primers (all are shown 5' to 3').

Primer name.	Sequence.
PetPro	TAATACGACTCACTATAGGG
PetTerm	GTGGCAGCAGCCAACTCAGC
BamA1130F	GAAATGCGTCAGATGGAAGGTGC
BamA1372R	TCGTTTTTGGTCCCGTTGATACCAAC
6HisBamA	GGGGGGCTAGCCACCACCACCACCACCACGCGGCGGAAGGTTTCGTTGTTAAAGACATC
PDΔ1	GGGGGGCTAGCGAACGTCCGACCATCGCGTCTATC
PDΔ2	CTGCTGGTTCAGGTTAAAGAACGTGTTTCTGCGGAAATCCAGCAGATC
PDΔ3	CCTGAAACTGGTTTTCCAGGAAGGTGCGCAGTACAAACTGTCTGGTGTTGAAG
PDΔ4	CGTTACCGTGAACATCACCGAAGGTGCGCGTTTCTACGTTCGTAAAATCCG
PDΔ5	CCCGGATCCGGTGTTACCCGCGTCCACGTTCACACGC
ΔL3	CGTCTGTTCTACAACGACTTCGGTGGTGGTGACTACACCAACAAATCTTACGG
ΔL4	CTGTCTAACATGCAGCCGCAGGGTGGTGGTTCTTTCAAAACCGACGACTTCACC
ΔL6	CGAAAACTTCTACGCGGGTGGTGTTTACTTCCCGCACCAGGCG
ΔL7	CTGGGACATGGGCACCGTTTGGGGTGGTGGTTCTAACATCCGTATGTCTGCG
ΔL8	GTTTTCTCTTACGCGCAGCCGTTCGGTGGTGGTGAACAGTTCCAGTTCAACATC
BamHIFw	CACCGGATCCTTCAACTTCGGTATCGG
XhoIRev	CCCCTCGAGAGATCTGCCGCCCCAAGTCTTGCCG
L2HARev	CAGTTCCGCGTAGGTCTGGTAGTAATCTGGTACATCATACGGATATTCGTCGTTTTTGGTGCCGTTGA
L3HARev	GTGTAGTCAGACAGGTCCGCGTAATCTGGTACATCATACGGATATTCGTCGTCCGCCTGGAAGTCGTT
L4HARev	GGTAGACGGGTGTTCACCGTAATCTGGTACATCATACGGATATTCCATAGAGTACAGGTAACGCC
L5HARev	GTAGTATTCGTTGTCAGAACCGTAATCTGGTACATCATACGGATATTCCGGAATGGTAACTTTGCCGG
L6HAFw	GGTGGTAAAGAAATGCCGTTCGAATATCCGTATGATGTACCAGATTACTACGAAAACTTCTACGCGGG
L7HAFw	ACCAACTGGGACTCTTCTCAGTATGAATATCCGTATGATGTACCAGATTACTCTGGTTACCCGGACTACTCTG
L8HAFw	GCCGTTCAAAAAATACGACGAATATCCGTATGATGTACCAGATTACGGTGACAAAGCGGAACAGTTC
β1HAFw	GGGGGATCCTTCAACGAATATCCGTATGATGTACCAGATTACTTCGGTATCGGTTACGGCACCGAATCTGG
β2HARev	GTTGTCCTGCTGAACACCCGCGTAATCTGGTACATCATACGGATATTCCTGGAAAGAAACACCAGATTCG
β3HARev	GGTAGTCGTTTTTGGTGCCGTTGTAATCTGGTACATCATACGGATATTCGATACCAACCGCGTAACCG
β4HARev	GAAGTACGGGTTGGTAACGTAATCTGGTACATCATACGGATATTCAGACAGTTCCGCGTAGGTCTGG
β5HARev	GTCGTTGTAGAACAGACGACCGTAATCTGGTACATCATACGGATATTCACCCAGAGAAACACCGTC
β6HARev	GGAAACCCAGGGTAACGTCGGTGTAATCTGGTACATCATACGGATATTCGCCGTAAGATTTGTTGGTGTA

β7HARev	GTTAGACAGAGAGTTGTGAACGTAATCTGGTACATCATACGGATATTCGTAACCCAGACCCGCACGCAG
β8HARev	TTGTTGTAGGTCCAACCGTAGTAATCTGGTACATCATACGGATATTCGTTGAAGGTGAAGTCGTCGGTTTT
β9HARev	GAATGGTAACTTTGCCGGTCAGGTAATCTGGTACATCATACGGATATTCGTTCACACGAGAACCGTC
β10HARev	CAATCGGAACGTAGGTCGCGGTGTAATCTGGTACATCATACGGATATTCGTCCAGGGTAACTTTGTAGTA
β11HARev	ACCGTCACCGTAACCCCAACGGTAATCTGGTACATCATACGGATATTCGGTACGACCCAGAACAACCCA
β12HAFw	AACGCGATGGCGGTTGCGTCTGAATATCCGTATGATGTACCAGATTACCTGGAATTTATCACCCCGAC
β13HAFw	CTCTGTTCGTACCTCTTTCTTCGAATATCCGTATGATGTACCAGATTACTGGGACATGGGCACCGTTTGG
β14HAFw	AACATCCGTATGTCTGCGGGTGAATATCCGTATGATGTACCAGATTACATCGCGCTGCAATGGATGTC
β15HAFw	CCGCTGGGGCCCCTGGTTTTCGAATATCCGTATGATGTACCAGATTACTCTTACGCGCAGCCGTTCAA
β16HAFw	GCGGAACAGTTCCAGTTCAACGAATATCCGTATGATGTACCAGATTACATCGGTAAAACCTGGTAATGAGG
