# Supplementary material for: Mutational and Topological Analysis of the Escherichia coli BamA Protein
Source: PLoS One. 2013 Dec 23;8(12):e84512. doi: 10.1371/journal.pone.0084512 (PMC3871556; doi:10.1371/journal.pone.0084512)
Supplement: Table S3 — Linker scanning insertions in BamA. (PDF) [file pone.0084512.s003.pdf]

Table S3. Linker scanning insertions in BamA.

Location of 5 amino acid insertions within BamA.	BamA structure affected.	Growth in absence of arabinose. [a)]	Maximum vancomycin concentration allowing growth. [b)]
pET17b	n/a	-	-
bamA	n/a	+	150
A2	Signal Sequence	+	37.5
I8	Signal Sequence	+	150
L12[c)]	Signal Sequence	+/-	0
L12 [c)]	Signal Sequence	+	150
S15	Signal Sequence	+	37.5
H30	POTRA 1	+	150
F31	POTRA 1	+	37.5
P47	POTRA 1	+	150
T69	POTRA 1	+	75
K89	POTRA 1	+	37.5
I97	POTRA 2	+	75
S100	POTRA 2	+	75
G118	POTRA 2	+	150
L137	POTRA 2	+	37.5
F140	POTRA 2	-	-
Y141	POTRA 2	+/-	0
V144	POTRA 2	+	37.5
G145	POTRA 2	+	0
K146	POTRA 2	+	0
T156	POTRA 2	+	75
L158	POTRA 2	+	75
Q170	POTRA 2	+	0
S174	POTRA 3	+	37.5
N181	POTRA 3	-	-
G184	POTRA 3	+	150
S195	POTRA 3	+	150
L199	POTRA 3	+	75
L231	POTRA 3	+/-	0
R237	POTRA 3	+/-	0
P249	POTRA 3	+	75
T257	POTRA 3	-	-
G293	POTRA 4	+	150
N297	POTRA 4	+	75
G298	POTRA 4	+	75
Y315	POTRA 4	+	75
Y317	POTRA 4	+	37.5
R321	POTRA 4	+	75
M325	POTRA 4	+	75
N329	POTRA 4	+	75
D332	POTRA 4	+	75
N340	POTRA 4	+	75
A343	POTRA 4	+	75
F354	POTRA 5	+	75

Table S3. Continued

Location of 5 amino acid insertion in BamA.	BamA secondary structure affected.	Growth in absence of arabinose. [a)]	Maximum vancomycin concentration allowing growth. [b)]
M372	POTRA 5	+	37.5
Q384	POTRA 5	+	37.5
D399	POTRA 5	+	75
G437	Loop 1	+	37.5
Q441	Beta 2	-	-
G443	Beta 2	-	-
G457	Beta 3	+	37.5
N459	Beta 3	+	0
Q466	Loop 2/ Beta 4	-	-
V480	Turn 2	+	150
D503	Loop 3/ Beta 6	-	-
Y504	Beta 6	+	37.5
Y509	Beta 6	-	-
G516	Beta 6	+	0
P518	Turn 3	+	75
L529	Beta 7	+	0
W546	Loop 4	+	0
P556	Loop 4	+	150
Y574	Beta 8	-	-
G575	Beta 8	+	37.5
Y578	Beta 8	+	37.5
I601	Loop 5	+	75
L613	Beta 10	-	-
G638	Beta 11/ Loop 6	+	75
G643	Loop 6	+	75
G656	Loop 6	+	75
V660	Loop 6	+	75
Q664	Loop 6	+/-	0
N666	Loop 6	-	-
P676	Loop 6	+	75
P684	Loop 6	+	150
D685	Loop 6	+	75
Y686	Loop 6	+	150
A714	Beta 12	-	-
P723	Turn 6	+	37.5
S726	Turn 6	+	75
D727	Turn 6	+	75
R734	Beta 13	+	0
P758	Loop 7	+	150
A770	Beta 14	-	-
Q789	Beta 15	-	-
G796	Loop 8	+	37.5
A799	Loop 8	-	-
W810	Beta 16	+	75

[a] The growth of the depletion strain JWD3, carrying either pET17b, pET17b/ *bamA* or pET17b/ *bamA* containing various insertions in *bamA*, was investigated by streaking bacteria onto nutrient agar plates supplemented with only 100 μg ml^{-1} ampicillin. Plates were incubated overnight at 37 ºC and strains were scored as follows: +, normal growth; +/-, weak growth; -, no growth. The results shown were determined from three independent experiments.

[b] JWD3 cells carrying pET17b/ *bamA*, containing various insertions in *bamA*, were struck out onto LB agar plates supplemented with 100 μg ml^{-1} ampicillin and 0, 37.5, 75 and 150 μg ml^{-1} vancomycin. Plates were incubated overnight at 37 ºC. No growth on plates without vancomycin is indicated as -. The results shown were determined from three independent experiments.

[c] Due to the location of the insertion site these two constructs have different amino acid insertions after position L12.
