# Supplementary material for: Mutational and Topological Analysis of the Escherichia coli BamA Protein
Source: PLoS One. 2013 Dec 23;8(12):e84512. doi: 10.1371/journal.pone.0084512 (PMC3871556; doi:10.1371/journal.pone.0084512)
Supplement: Table S4 — 6His tagged BamA insertions. (PDF) [file pone.0084512.s004.pdf]

Table S4. 6His tagged BamA insertions.

Location of 5 amino acid insertions in BamA.	BamA secondary structure affected.	Growth in absence of arabinose. [a)]	Maximum vancomycin concentration allowing growth. [b)]
pET17b	n/a	-	-
6hisbamA	n/a	+	150
K89	POTRA 1	+/-	0
F140	POTRA 2	-	nd
Y141	POTRA 2	+	0
Q170	POTRA 2	+	0
N181	POTRA 3	-	nd
L231	POTRA 3	-	nd
R237	POTRA 3	-	nd
T257	POTRA 3	-	nd
Y317	POTRA 4	+	37.5
Q384	POTRA 5	+	37.5
Q441	Beta 2	-	nd
Q466	Loop 2/ Beta 4	-	nd
D503	Loop 3/ Beta 6	-	nd
Y509	Beta 6	-	nd
W546	Loop 4	-	nd
Y574	Beta 8	-	nd
L613	Beta 10	-	nd
Q664	Loop 6	-	nd
N666	Loop 6	-	nd
A714	Beta 12	-	nd
A770	Beta 14	-	nd
Q789	Beta 15	-	nd
A799	Loop 8	-	nd

[a)] The growth of the depletion strain JWD3, carrying either pET17b, pET17b/ *6hisbamA* or pET17b/ *6hisbamA* containing various insertions in *bamA*, was investigated by streaking bacteria onto nutrient agar plates supplemented with only 100 μg ml^{-1} ampicillin. Plates were incubated overnight at 37 ^{o}C and strains were scored as follows: +, normal growth; +/-, weak growth; -, no growth. The results shown were determined from three independent experiments.

[b)] JWD3 cells containing version of pET17b/ *6hisbamA*, which enabled cells to grow in the absence of arabinose, were struck onto LB agar plates supplemented with 100 μg ml^{-1} ampicillin and 0, 37.5, 75 and 150 μg ml^{-1} vancomycin. Plates were incubated overnight at 37 ^{o}C. Constructs that were not tested are indicated as nd (not determined). The results shown were determined from three independent experiments.
