# Supplementary material for: Mutational and Topological Analysis of the Escherichia coli BamA Protein
Source: PLoS One. 2013 Dec 23;8(12):e84512. doi: 10.1371/journal.pone.0084512 (PMC3871556; doi:10.1371/journal.pone.0084512)
Supplement: Table S5 — HA insertions in BamA. (PDF) [file pone.0084512.s005.pdf]

Table S5. HA insertions in BamA.

Location of HA epitopes within BamA.	BamA structure affected.	Growth in absence of arabinose. [a)]	Maximum vancomycin concentration allowing growth. [b)]
pET17b	n/a	-	-
bamA	n/a	+	150
D464	Loop 2	-	-
D498	Loop 3	+/-	0
M552	Loop 4	+	150
P602	Loop 5	+	0
F648	Loop 6	-	-
Y754	Loop 7	+	150
D795	Loop 8	-	-
N427	Beta 1	-	-
Q441	Beta 2	-	-
I458	Beta 3	-	-
S472	Beta 4	-	-
G486	Beta 5	+/-	0
G510	Beta 6	-	-
Y531	Beta 7	-	-
N573	Beta 8	-	-
N594	Beta 9	-	-
D614	Beta 10	-	-
T633	Beta 11	-	-
S715	Beta 12	-	-
F738	Beta 13	-	-
G771	Beta 14	-	-
F785	Beta 15	-	-
N805	Beta 16	-	-

[a)] The growth of the depletion strain JWD3, carrying either pET17b, pET17b/ *bamA* or pET17b/ *bamA* containing various HA epitope insertions in *bamA*, was investigated by streaking bacteria onto nutrient agar plates supplemented with only 100 μg ml^{-1} ampicillin. Plates were incubated overnight at 37 ºC and strains were scored as follows: +, normal growth; +/-, weak growth; -, no growth. The results shown were determined from three independent experiments. The HA epitope encodes for the peptide sequence YPYDVPDYA.

[b)] JWD3 cells carrying pET17b/ *bamA*, containing various HA insertions in *bamA*, were struck out onto LB agar plates supplemented with 100 μg ml^{-1} ampicillin and 0, 37.5, 75 and 150 μg ml^{-1} vancomycin. Plates were incubated overnight at 37 ºC. No growth on plates without vancomycin is indicated as -. The results shown were determined from three independent experiments.
