# Supplementary material for: Mutational and Topological Analysis of the Escherichia coli BamA Protein
Source: PLoS One. 2013 Dec 23;8(12):e84512. doi: 10.1371/journal.pone.0084512 (PMC3871556; doi:10.1371/journal.pone.0084512)
Supplement: Table S6 — 6His tagged BamA barrel loop deletions. (PDF) [file pone.0084512.s006.pdf]

Table S6. 6His tagged BamA barrel loop deletions.

Loop deleted.	Growth in absence of arabinose. [a)]	Maximum vancomycin concentration allowing growth. [b)]
pET17b	-	-
6hisbamA	+	150
ΔL3	+	75
ΔL4	-	nd
ΔL6	-	nd
ΔL7	+/-	0
ΔL8	+	0

[a)] Growth of the depletion strain JWD3, carrying either pET17b, pET17b/ *6hisbamA* or pET17b/ *6hisbamA* containing various loop deletions within *bamA*, was investigated by streaking bacteria onto nutrient agar plates supplemented with only 100 μg ml^{-1} ampicillin. Plates were incubated overnight at 37 ºC and strains were scored as follows: +, normal growth; +/-, weak growth; -, no growth. The results shown were determined from three independent experiments.

[b)] JWD3 cells containing the versions of pET17b/ *6hisbamA*, which enabled cells to grow in the absence of arabinose, were struck out onto LB agar plates supplemented with 100 μg ml^{-1} ampicillin and 0, 37.5, 75 and 150 μg ml^{-1} vancomycin. Plates were incubated overnight at 37 ºC. Constructs that were not tested are indicated as nd (not determined). The results shown were determined from three independent experiments.
