# Supplementary material for: Mutational and Topological Analysis of the Escherichia coli BamA Protein
Source: PLoS One. 2013 Dec 23;8(12):e84512. doi: 10.1371/journal.pone.0084512 (PMC3871556; doi:10.1371/journal.pone.0084512)
Supplement: Figure S1 — DNA sequence of bamA. (PDF) [file pone.0084512.s007.pdf]

Fig. S1.

CATATGGCGATGAAAAAACTGCTGATCGCGTCTCTGCTGTTCTCTTCTGCGACCGTTTACGGT**GCTAG**
CGAAGGTTTCGTTGTTAAAGACATCCACTTTGAAGGTCTGCAACGTGTTGCGGTTGGTGCGGCGCTGC
TGTCTATGCCGGTTCGTACCGGCGACACCGTGAACGACGAAGACATCTCTAACACCATCCGTGCGCTG
TTCGCGACCGGCAACTTTGAAGACGTTCGTGTTCTGCGTGACGGTGACACCCTGCTGGTTCAGGTTAA
AGAACGTCCGACCATCGCGTCTATCACCTTCTCTGGTAACAAATCTGTTAAAGACGACATGCTGAAAC
AGAACCTGGAAGCGTCTGGTGTTCGTGTTGGTGAATCTCTGGACCGTACCACCATCGCGGACATCGAA
AAAGGTCTGGAAGACTTCTACTACTCTGTTGGTAAATACTCTGCGTCTGTTAAAGCGGTTGTTACCCC
GCTGCCGCGTAACCGTGTTGACCTGAAACTGGTTTTCCAGGAAGGTGTTTCTGCGGAAATCCAGCAGA
TCAACATCGTTGGTAACCACGCTTTCACCACCGACGAACTGATCTCTCACTTCCAACTGCGTGACGAA
GTTCCGTGGTGGAACGTGGTTGGTGACCGTAAATACCAGAAACAGAAACTGGCGGGTGACCTGGAAAC
CCTGCGTTCTTACTACCTGGACCGTGGTTACGCGCGTTTCAACATCGACTCTACCCAGGTTTCTCTGA
CCCCGGACAAAAAAGGTATCTACGTTACCGTGAACATCACCGAAGGTGACCAGTACAAACTGTCTGGT
GTTGAAGTTTCTGGTAACCTGGCGGGTCACTCTGCGGAAATCGAACAACTGACCAAAATCGAACCGGG
TGAACTGTATAACGGCACCAAAGTTACCAAAATGGAAGACGACATCAAAAAACTGCTGGGTCGTTACG
GTTACGCTTACCCGCGTGTTCAGTCTATGCCGGAAATCAACGACGCGGACAAAACCGTTAAACTGCGT
GTGAACGTGGACGCGGGTAACCGTTTCTACGTTCGTAAAATCCGTTTTGAAGGTAACGACACCTCTAA
AGACGCGGTTCTGCGTCGTGAAATGCGTCAGATGGAAGGTGCGTGGCTGGGTTCTGACCTGGTTGACC
AGGGTAAAGAACGTCTGAACCGTCTGGGTTTCTTTGAAACCGTTGACACCGACACCCAGCGTGTTCCG
GGTTCCCCGGACCAGGTTGACGTTGTTTACAAAGTTAAAGAACGTAACACC**GGATCC**TTCAACTTCGG
TATCGGTTACGGCACCGAATCTGGTGTTTCTTTCCAGGCGGGTGTTCAGCAGGACAACTGGCTGGGCA
CCGGTTACGCGGTTGGTATCAACGGCACCAAAAACGACTACCAGACCTACGCGGAACTGTCTGTTACC
AACCCGTACTTCACCGTTGACGGTGTTTCTCTGGGTGGTCGTCTGTTCTACAACGACTTCCAGGCGGA
CGACGCGGACCTGTCTGACTACACCAACAAATCTTACGGCACCGACGTTACCCTGGGTTTCCCGATCA
ACGAATACAACTCTCTGCGTGCGGGTCTGGGTTACGTTCACAACTCTCTGTCTAACATGCAGCCGCAG
GTTGCGATGTGGCGTTACCTGTACTCTATGGGTGAACACCCGTCTACCTCTGACCAGGACAACTCTTT
CAAAACCGACGACTTCACCTTCAACTACGGTTGGACCTACAACAAACTGGACCGTGGTTACTTCCCGA
CCGACGGTTCTCGTGTGAACCTGACCGGCAAAGTTACCATTCCGGGTTCTGACAACGAATACTACAAA
GTTACCCTGGACACCGCGACCTACGTTCCGATTGACGACGACCACAAATGGGTTGTTCTGGGTCGTAC
CCGTTGGGGTTACGGTGACGGTCTGGGTGGTAAAGAAATGCCGTTCTACGAAAACTTCTACGCGGGTG
GTTCTTCTACCGTTCGTGGTTTCCAGTCTAACACCATCGGTCCGAAAGCGGTTTACTTCCCGCACCAG
GCGTCTAACTACGACCCGGACTACGACTACGAATGCGCGACCCAGGACGGTGCGAAAGACCTGTGCAA
ATCTGACGACGCGGTTGGTGGTAACGCGATGGCGGTTGCGTCTCTGGAATTTATCACCCCGACCCCGT
TCATCTCTGACAAATACGCGAACTCTGTTCGTACCTCTTTCTTCTGGGACATGGGCACCGTTTGGGAC
ACCAACTGGGACTCTTCTCAGTATTCTGGTTACCCGGACTACTCTGACCCGTCTAACATCCGTATGTC
TGCGGGTATCGCGCTGCAATGGATGTCTCCGCTGGGGCCCCTGGTTTTCTCTTACGCGCAGCCGTTCA
AAAAATACGACGGTGACAAAGCGGAACAGTTCCAGTTCAACATCGGTAAAACCTGGTAATGAGGGCCC
TTAGTGTTTAGCTATGCTCAACCTTTTAAGAAGTATGATGGCGATAAGGCTGAGCAATTTCAATTTAA
TATCGGCAAGACTTGGGGCGGCAGATCT**CTCGAG**

Figure S1. DNA sequence of *bamA*. The figure show the DNA sequence of codon optimized *bamA* used in this study. The restriction enzyme recognition sites located within wild-type *E. coli bamA* were purged from the sequence and *Nde*I, *Nhe*I, *Bam*HI and *Xho*I were introduced at the positions indicated (shown as bold and underlined).
