# Supplementary material for: Mutational and Topological Analysis of the Escherichia coli BamA Protein
Source: PLoS One. 2013 Dec 23;8(12):e84512. doi: 10.1371/journal.pone.0084512 (PMC3871556; doi:10.1371/journal.pone.0084512)
Supplement: Figure S2 — Alignment of the BamA sequences. (PDF) [file pone.0084512.s008.pdf]

Fig. S2.

```
BamA            MAMKKLLIASLLFSSATVYGA-EGFVVKDIHFEGLQRVAVGAALLSMPVRTGDTVNDEDI
BamA opt        MAMKKLLIASLLFSSATVYGASEGFVVKDIHFEGLQRVAVGAALLSMPVRTGDTVNDEDI
                ********************* **************************************

BamA            SNTIRALFATGNFEDVRVLRDGDTLLVQVKERPTIASITFSGNKSVKDDMLKQNLEASGV
BamA opt        SNTIRALFATGNFEDVRVLRDGDTLLVQVKERPTIASITFSGNKSVKDDMLKQNLEASGV
                ************************************************************

BamA            RVGESLDRTTIADIEKGLEDFYYSVGKYSASVKAVVTPLPRNRVDLKLVFQEGVSAEIQQ
BamA opt        RVGESLDRTTIADIEKGLEDFYYSVGKYSASVKAVVTPLPRNRVDLKLVFQEGVSAEIQQ
                ************************************************************

BamA            INIVGNHAFTTDELISHFQLRDEVPWWNVVGDRKYQKQKLAGDLETLRSYYLDRGYARFN
BamA opt        INIVGNHAFTTDELISHFQLRDEVPWWNVVGDRKYQKQKLAGDLETLRSYYLDRGYARFN
                ************************************************************

BamA            IDSTQVSLTPDKKGIYVTVNITEGDQYKLSGVEVSGNLAGHSAEIEQLTKIEPGELYNGT
BamA opt        IDSTQVSLTPDKKGIYVTVNITEGDQYKLSGVEVSGNLAGHSAEIEQLTKIEPGELYNGT
                ************************************************************

BamA            KVTKMEDDIKKLLGRYGYAYPRVQSMPEINDADKTVKLRVNVDAGNRFYVRKIRFEGNDT
BamA opt        KVTKMEDDIKKLLGRYGYAYPRVQSMPEINDADKTVKLRVNVDAGNRFYVRKIRFEGNDT
                ************************************************************

BamA            SKDAVLRREMRQMEGAWLGSDLVDQGKERLNRLGFFETVDTDTQRVPGSPDQVDVVYKVK
BamA opt        SKDAVLRREMRQMEGAWLGSDLVDQGKERLNRLGFFETVDTDTQRVPGSPDQVDVVYKVK
                ************************************************************

BamA            ERNTGSFNFGIGYGTESGVSFQAGVQQDNWLGTGYAVGINGTKNDYQTYAELSVTNPYFT
BamA opt        ERNTGSFNFGIGYGTESGVSFQAGVQQDNWLGTGYAVGINGTKNDYQTYAELSVTNPYFT
                ************************************************************

BamA            VDGVSLGGRLFYNDFQADDADLSDYTNKSYGTDVTLGFPINEYNSLRAGLGYVHNSLSNM
BamA opt        VDGVSLGGRLFYNDFQADDADLSDYTNKSYGTDVTLGFPINEYNSLRAGLGYVHNSLSNM
                ************************************************************

BamA            QPQVAMWRYLYSMGEHPSTSDQDNSFKTDDFTFNYGWTYNKLDRGYFPTDGSRVNLTGKV
BamA opt        QPQVAMWRYLYSMGEHPSTSDQDNSFKTDDFTFNYGWTYNKLDRGYFPTDGSRVNLTGKV
                ************************************************************

BamA            TIPGSDNEYYKVTLDTATYVPIDDDHKWVVLGRTRWGYGDGLGGKEMPFYENFYAGGSST
BamA opt        TIPGSDNEYYKVTLDTATYVPIDDDHKWVVLGRTRWGYGDGLGGKEMPFYENFYAGGSST
                ************************************************************

BamA            VRGFQSNTIGPKAVYFPHQASNYDPDYDYECATQDGAKDLCKSDDAVGGNAMAVASLEFI
BamA opt        VRGFQSNTIGPKAVYFPHQASNYDPDYDYECATQDGAKDLCKSDDAVGGNAMAVASLEFI
                ************************************************************

BamA            TPTPFISDKYANSVRTSFFWDMGTVWDTNWDSSQYSGYPDYSDPSNIRMSAGIALQWMSP
BamA opt        TPTPFISDKYANSVRTSFFWDMGTVWDTNWDSSQYSGYPDYSDPSNIRMSAGIALQWMSP
                ************************************************************

BamA            LGPLVFSYAQPFKKYDGDKAEQFQFNIGKTW
BamA opt        LGPLVFSYAQPFKKYDGDKAEQFQFNIGKTW
                *******************************
```

Figure S2. Alignment of the BamA sequences. The figure shows the alignment of the wild-type *E. coli* BamA protein sequence with the codon optimized version of BamA used in the study (BamA opt). Note that the introduction of a *Nhe*I site into the codon optimized version of *bamA* introduces an addition serine residue after the end of the BamA signal sequence at Ala21. This addition does not affect the activity of BamA.
