# Supplementary material for: Mutational and Topological Analysis of the Escherichia coli BamA Protein
Source: PLoS One. 2013 Dec 23;8(12):e84512. doi: 10.1371/journal.pone.0084512 (PMC3871556; doi:10.1371/journal.pone.0084512)
Supplement: Figure S3 — Depletion of BamA in E. coli JWD3 cells. (PDF) [file pone.0084512.s009.pdf]

**Fig. S3.**

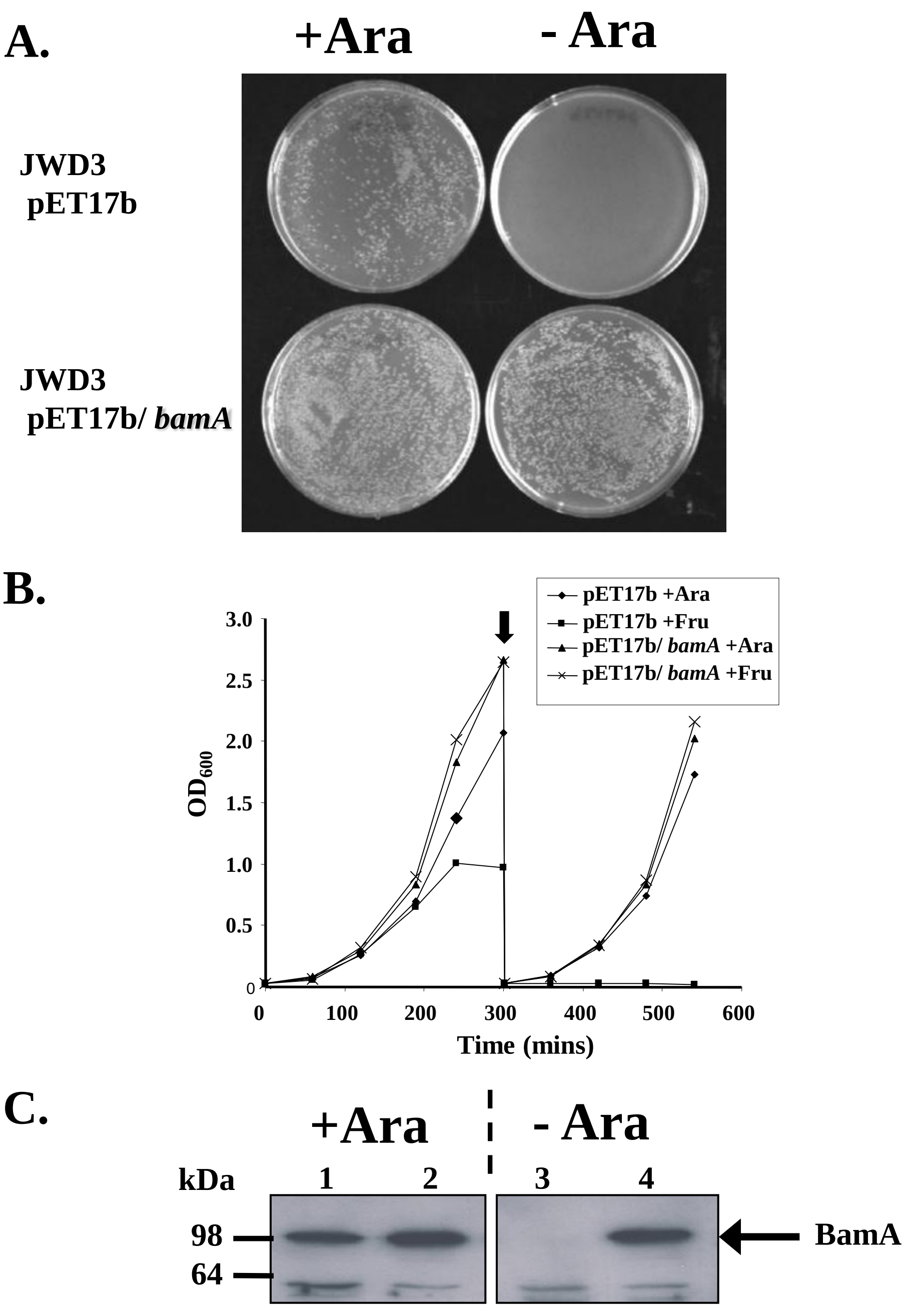


**Figure S3. Depletion of BamA in *E. coli* JWD3 cells.** (A) JWD3 cells transformed with pET17b or pET17b/ *bamA* were plated on to nutrient agar plates in the presence or absence of arabinose (+Ara or –Ara). (B) BamA depletion analysis. The panel shows the growth of JWD3 cells carrying pET17b and pET17b/ *bamA* in 50 ml of Lennox broth in the presence of 0.05% arabinose or fructose (+Ara or + Fru). Optical density ($OD_{600}$) was monitored over time and after 300 minutes cultures were sampled and subcultured into fresh medium (indicated by arrows). (C) A Western blot of total cell protein from JWD3 cells carrying pET17b or pET17b/ *bamA*. Blots were probed with anti-BamA POTRA domain antiserum and the SDS PAGE gel was loaded as follows: lane 1, JWD3 pET17b cells grown with arabinose; lane 2, JWD3 pET17b/ *bamA* cells grown with arabinose; lane 3, JWD3 pET17b cells without arabinose; lane 4, JWD3 pET17b/ *bamA* cells without arabinose. The image displayed comes from samples loaded onto the same SDS PAGE gel and Western blot. The location of BamA is indicated by arrows.
