# Supplementary material for: Mutational and Topological Analysis of the Escherichia coli BamA Protein
Source: PLoS One. 2013 Dec 23;8(12):e84512. doi: 10.1371/journal.pone.0084512 (PMC3871556; doi:10.1371/journal.pone.0084512)
Supplement: Figure S4 — Location of 5 amino acid insertions within the POTRA domains of BamA. (PDF) [file pone.0084512.s010.pdf]

Fig. S4.

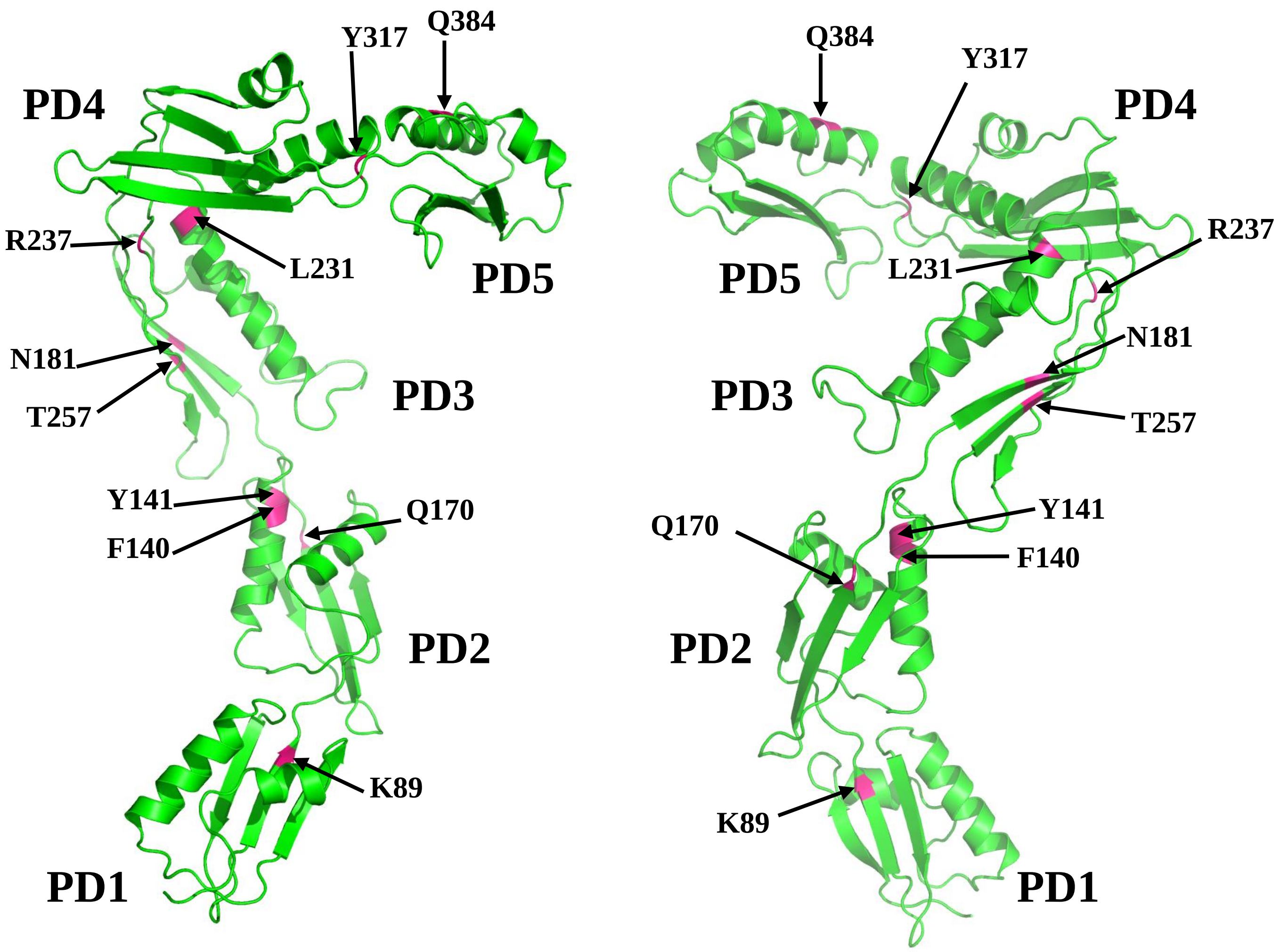

Figure S4. Location of 5 amino acid insertions within the POTRA domains of BamA. The figure shows a model displaying the five POTRA domains of BamA (PD1 to PD5) and the location of 5 amino acid insertions (K89, F140, Y141, Q170, N181, L231, R237, T257, Y317 and Q384), which were isolated by linker scanning mutagenesis. N-terminal 6His tags were introduced into these mutant constructs to enable detection using Western blotting (Fig. 1). The model was generated using PyMol [34] by combining the available crystal structures of BamA $POTRA_1$ to $POTRA_4$ (3ECF) [8] and $POTRA_4$ and $POTRA_5$ (3OG5) [10].
