# Supplementary material for: Mutational and Topological Analysis of the Escherichia coli BamA Protein
Source: PLoS One. 2013 Dec 23;8(12):e84512. doi: 10.1371/journal.pone.0084512 (PMC3871556; doi:10.1371/journal.pone.0084512)
Supplement: Figure S5 — Pull down experiments with 6HisBamA proteins carrying 5 amino acid insertions. (PDF) [file pone.0084512.s011.pdf]

Fig. S5.

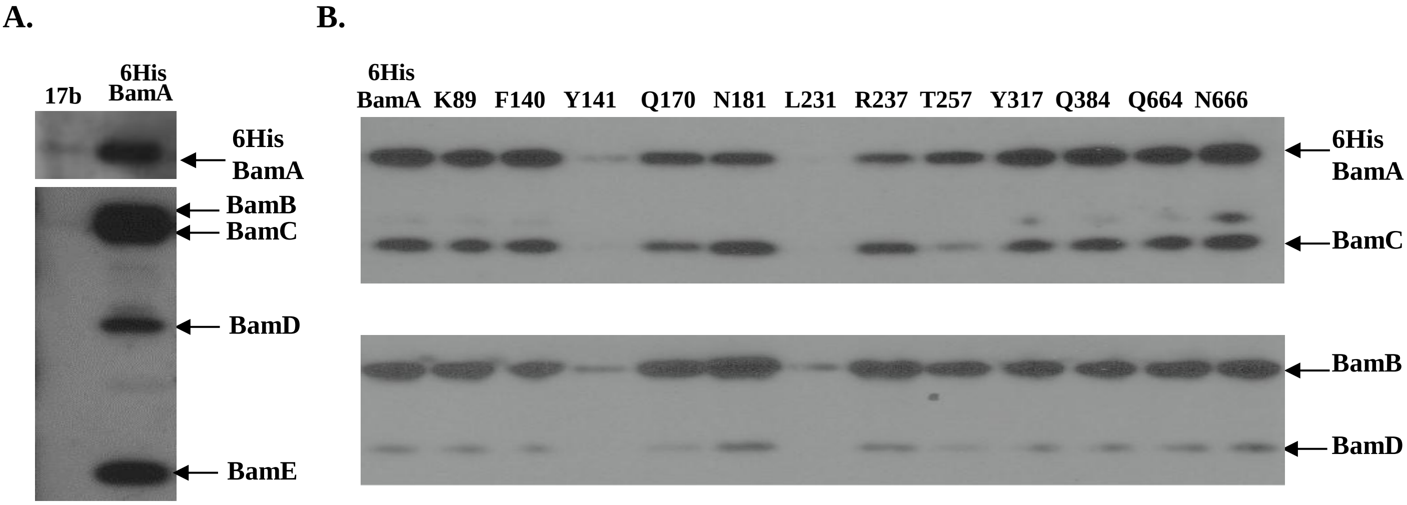

Figure S5. Pull down experiments with 6HisBamA proteins carrying 5 amino acid insertions. N-terminal 6His tags were introduced into K89, F140, Y141, Q170, N181, L231, R237, T257, Y317, Q384, Q664 and N666 insertion constructs, cloned into pET17b, and each plasmid was transformed into strain BL21(DE3). Cells were grown to an OD600 of ~0.8 without induction of protein expression and proteins associated with each 6HisBamA protein were isolated by incubating cell lysates with NTA resin, as detailed in Materials and Methods. (A) Proteins eluted from NTA resin incubated with lysates from BL21(DE3) cells carrying either pET17b or pET17B/ *6hisbamA* were subjected to Western blotting. Blots were probed with anti-BamA, anti-BamB, anti-BamC, anti-BamD and anti-BamE antisera. (B) Proteins eluted from NTA resin incubated with lysates from BL21(DE3) cells carrying the various pET17B/ *6hisbamA* insertion constructs were subjected to Western blotting. Blots were probed using anti-6His antiserum and anti-BamB, anti-BamC and anti-BamD antisera. Samples were normalised to ensure that similar amounts of 6HisBamA were loaded. This was not possible for the Y141 and L231 insertion constructs, as 6HisBamA containing these insertions bound extremely poorly to the NTA resin. The location of 6HisBamA, BamB, BamC, BamD and BamE proteins are indicated.
