# Supplementary material for: Mutational and Topological Analysis of the Escherichia coli BamA Protein
Source: PLoS One. 2013 Dec 23;8(12):e84512. doi: 10.1371/journal.pone.0084512 (PMC3871556; doi:10.1371/journal.pone.0084512)
Supplement: Figure S6 — Depletion analysis of BamA POTRA domains on solid media. (PDF) [file pone.0084512.s012.pdf]

**Fig. S6.**

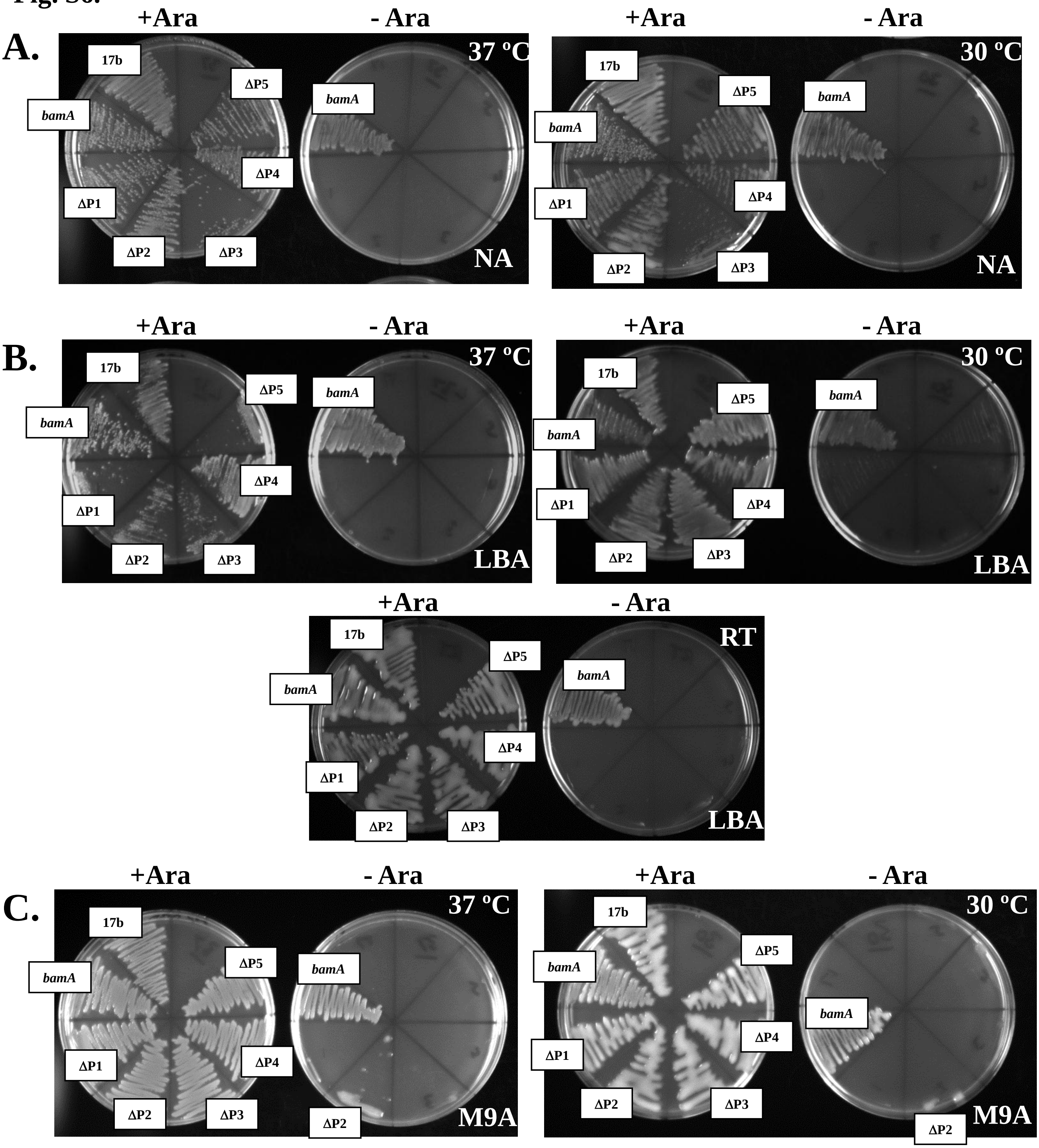


**Figure S6. Depletion analysis of BamA POTRA domains on solid media.** The figure shows the growth of JWD3 cells carrying pET17b, pET17b/ *bamA* or pET17b encoding *bamA* constructs with deletions of each POTRA domain (ΔP1 to ΔP5). Cells were struck out on to: (A) nutrient agar plates (NA), (B) LB agar plates (LBA) and (C) M9 medium agar plates supplemented with 0.2% glucose (M9A) in the presence or absence of 0.2 % arabinose (+Ara or –Ara). Cells grown on NA and LBA plates were incubated at 37 ºC and 30 ºC for 20 h, whilst for M9A plates incubation was extended to 40 h. In (B), an additional LBA agar plate was incubated at room temperature (RT) for 4 days.
