# Supplementary material for: Mutational and Topological Analysis of the Escherichia coli BamA Protein
Source: PLoS One. 2013 Dec 23;8(12):e84512. doi: 10.1371/journal.pone.0084512 (PMC3871556; doi:10.1371/journal.pone.0084512)
Supplement: Figure S7 — Alignment of the β-barrel domains from BamA and FhaC. (PDF) [file pone.0084512.s013.pdf]

Fig. S7.

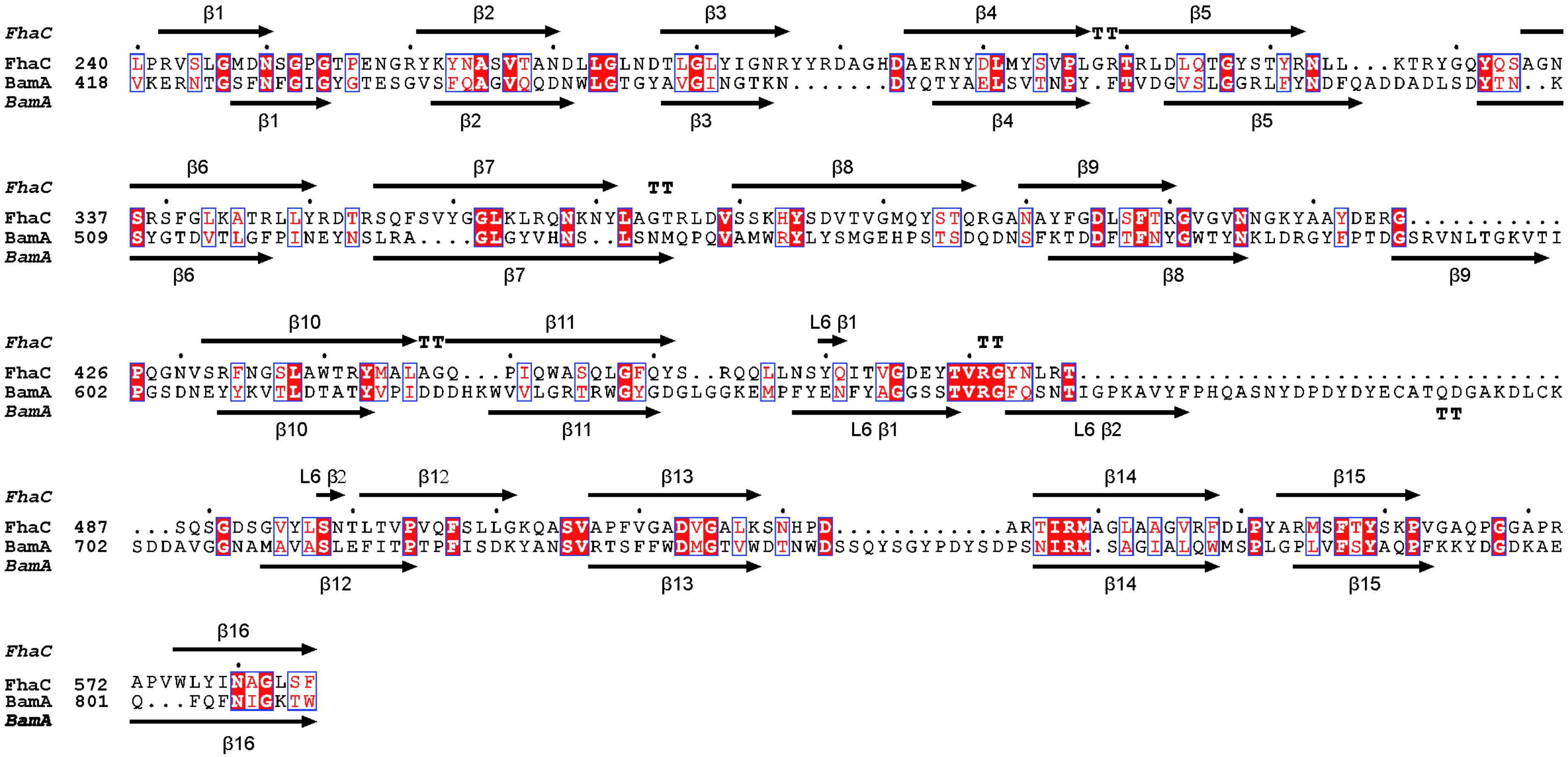

Figure S7. Alignment of the β-barrel domains from BamA and FhaC. The figure shows the alignment of the β-barrel domain of FhaC from *Bordetella pertussis* (GenBank CAA46092) with that of BamA from *E. coli* K-12 (GenBank AAC73288). Secondary structure for FhaC is derived from the crystal structure of FhaC (2QDZ) [13] and is shown above the alignment, whilst that for BamA is from our model predications and is displayed below (see Fig. 2). Predicted β-strands in loop L6 are labelled as L6 β1 and L6 β2.
