# Supplementary material for: Mutational and Topological Analysis of the Escherichia coli BamA Protein
Source: PLoS One. 2013 Dec 23;8(12):e84512. doi: 10.1371/journal.pone.0084512 (PMC3871556; doi:10.1371/journal.pone.0084512)
Supplement: Figure S8 — Comparison of the BamA and FhaC secondary structure surrounding loop L4 of the β-barrel domain. (PDF) [file pone.0084512.s014.pdf]

Fig. S8.

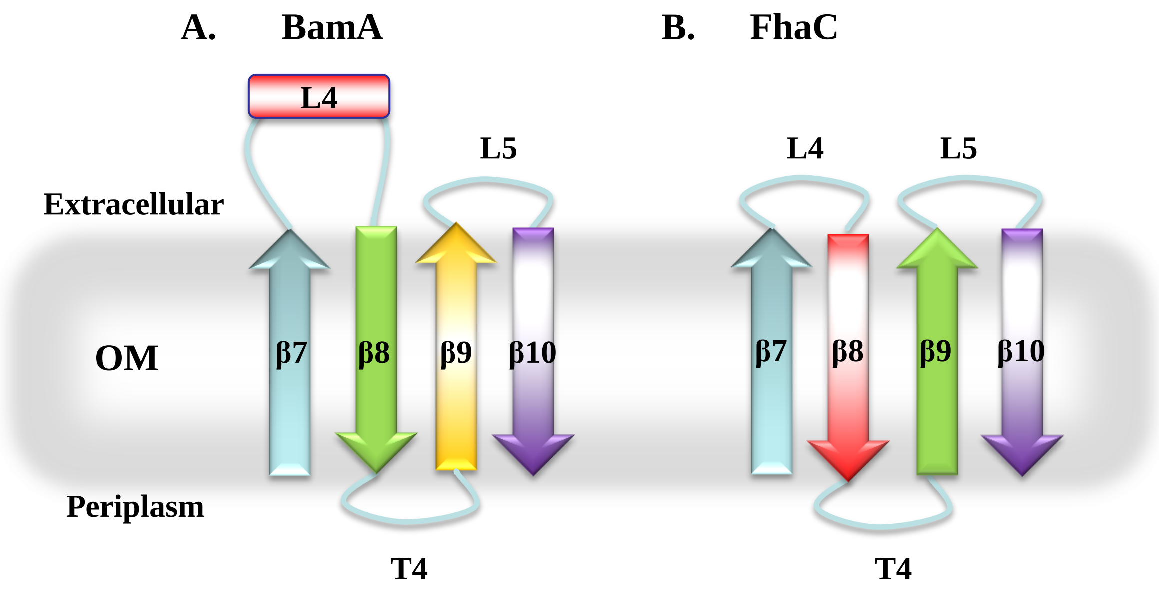

Figure S8. Comparison of the BamA and FhaC secondary structure surrounding loop L4 of the β-barrel domain. The figure shows the secondary structure of β-strands β7 to β10 for (A) BamA and (B) FhaC. The secondary structure for BamA it is derived from our model predications (see Fig. 2) and for FhaC it was taken the published crystal structure (2QDZ) [13]. β-strands are shown as arrows and are colour coded to represent sequence synteny. In panel (A) BamA β9 does not have an equivalent β-strand in FhaC and so is coloured differently.
