# Supplementary material for: Mutational and Topological Analysis of the Escherichia coli BamA Protein
Source: PLoS One. 2013 Dec 23;8(12):e84512. doi: 10.1371/journal.pone.0084512 (PMC3871556; doi:10.1371/journal.pone.0084512)
Supplement: Figure S9 — Alignment of BamA/Omp85 orthologues. (PDF) [file pone.0084512.s015.pdf]

Fig. S9.

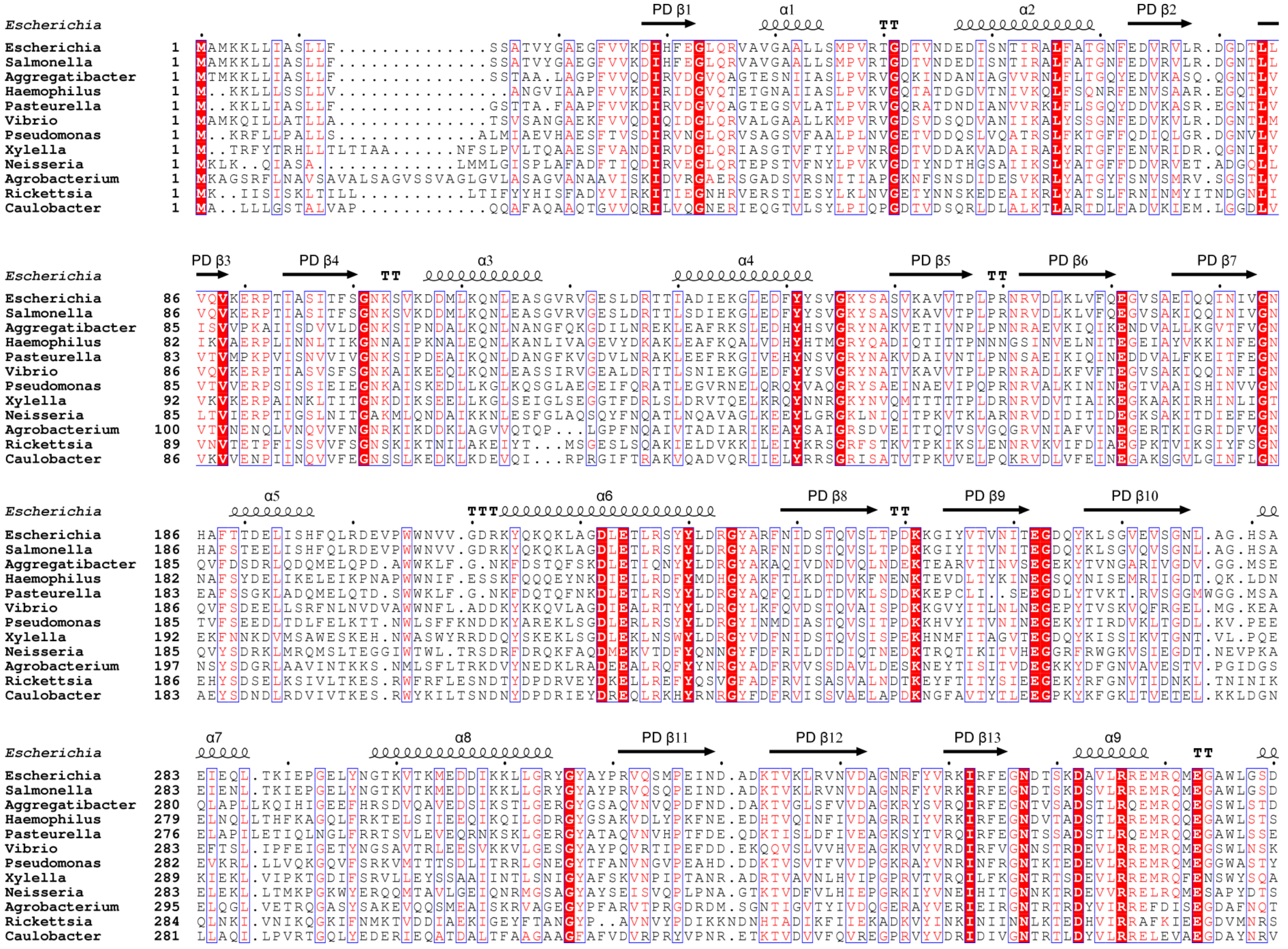

Escherichia
PD β1
α1
TT
α2
PD β2
Escherichia 1
Salmonella 1
Aggregatibacter 1
Haemophilus 1
Pasteurella 1
Vibrio 1
Pseudomonas 1
Xylella 1
Neisseria 1
Agrobacterium 1
Rickettsia 1
Caulobacter 1
PD β3
PD β4
TT
α3
α4
PD β5
TT
PD β6
PD β7
Escherichia 86
Salmonella 86
Aggregatibacter 85
Haemophilus 82
Pasteurella 83
Vibrio 86
Pseudomonas 85
Xylella 92
Neisseria 85
Agrobacterium 100
Rickettsia 89
Caulobacter 86
α5
TTT
α6
PD β8
TT
PD β9
PD β10
Escherichia 186
Salmonella 186
Aggregatibacter 185
Haemophilus 182
Pasteurella 183
Vibrio 186
Pseudomonas 185
Xylella 192
Neisseria 185
Agrobacterium 197
Rickettsia 186
Caulobacter 183
α7
α8
PD β11
PD β12
PD β13
α9
TT
Escherichia 283
Salmonella 283
Aggregatibacter 280
Haemophilus 279
Pasteurella 276
Vibrio 283
Pseudomonas 282
Xylella 289
Neisseria 283
Agrobacterium 295
Rickettsia 284
Caulobacter 281

Fig. S9. continued.

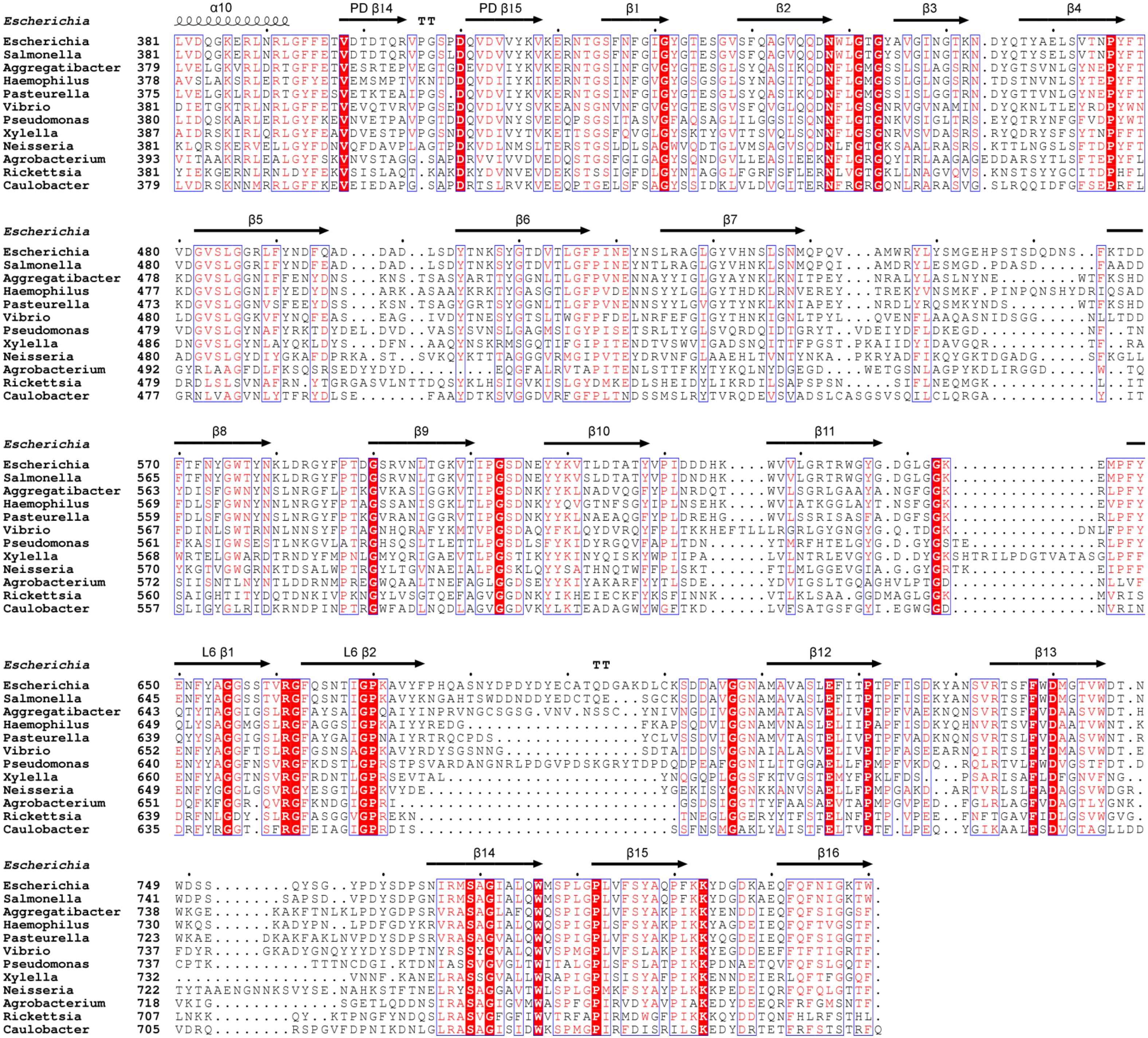

Figure S9. Alignment of BamA/ Omp85 orthologues. The figure shows the alignment of *E. coli* K-12 BamA (GenBank AAC73288) with the BamA/ Omp85 orthologues from *Salmonella enterica serovar* Typhi (GenBank CAD08682), *Aggregatibacter actinomycetemcomitans* (GenBank ACX81664), *Haemophilus ducreyi* (GenBank AAP96041), *Pasteurella multocida* (GenBank AAC44600), *Vibrio cholerae* (GenBank AAF95396), *Pseudomonas aeruginosa* (GenBank ABR83527), *Xylella fastidiosa* (GenBank AAF83856), *Neisseria gonorrhoeae* (GenBank ACF31073), *Agrobacterium tumefaciens* (WP003512849), *Rickettsia prowazekii* (GenBank CAA14627), *Caulobacter crescentus* (GenBank AAK23890). Secondary structure, derived from our model predications (Fig. 2), is indicated above the alignment. POTRA domain β-strands are designated PD β1 to PD β15, whilst those in the β-barrel are labelled β1 to β16. Predicted β-strands in loop L6 are labelled as L6 β1 and L6 β2.
